# Genetic deletion of ASIC3 alters left ventricular remodeling and autonomic function after myocardial infarction in mice

**DOI:** 10.14814/phy2.70823

**Published:** 2026-03-11

**Authors:** Karley M. Monaghan, David D. Gibbons, Chad C. Ward, Maram El‐Geneidy, William J. Kutschke, Kathy A. Zimmerman, Donald A. Morgan, Harald M. Stauss, Anne Marie S. Harding, Michelle C. M. Bader, Peter M. Snyder, Rasna Sabharwal, Robert M. Weiss, Kamal Rahmouni, Christopher J. Benson

**Affiliations:** ^1^ Department of Neuroscience and Pharmacology University of Iowa Iowa City Iowa USA; ^2^ Department of Internal Medicine University of Iowa Iowa City Iowa USA; ^3^ Department of Health, Sport, and Human Physiology University of Iowa Iowa City Iowa USA; ^4^ Veterans Affairs Health Care System Iowa City Iowa USA

**Keywords:** cardiac hypertrophy, ischemic heart disease, neurohormonal activation

## Abstract

Cardiac afferent neurons have been shown to trigger overactivation of neurohormonal systems known to drive adverse cardiac remodeling following myocardial infarction (MI). Acid‐sensing ion channels (ASICs) that are highly expressed in cardiac sympathetic afferents sense ischemia‐induced myocardial acidosis. We hypothesized that genetic deletion of ASICs might abrogate disadvantageous remodeling after MI by disrupting afferent signaling pathways otherwise resulting in overactivation of neurohormonal responses. To test this, we induced MI in wild type (WT) and ASIC3^−/−^ mice and assessed cardiac remodeling by serial echocardiography. We found that ASIC3^−/−^ mice had less LV dilation relative to ischemic zone fraction, increased LV mass and wall thickness, and increased stroke volume compared to WT mice after MI. To investigate a potential role of the autonomic nervous system, we measured renal and splanchnic sympathetic nerve activity (SNA), heart rate and systolic blood pressure variability (sBPV), and hemodynamic responses to atropine and propranolol. Following MI, ASIC3^−/−^ mice had lower baroreceptor‐renal SNA reflex sensitivity than WT mice, associated with elevated sBPV. Our data show that ASIC3 plays an important role in cardiac remodeling after MI potentially via modulation of baroreflex sensitivity and sBPV. ASIC3 may be further investigated as a potential therapeutic target in heart failure.

## 1 INTRODUCTION

Therapeutic advancements after myocardial infarction (MI) have led to remarkably improved outcomes. However, with better survival, more patients are going on to develop progressive left ventricular (LV) dysfunction and heart failure. In fact, ischemic heart disease is the leading cause of heart failure (Heidenreich et al., 2022). The development of heart failure after MI is determined by the initial infarct size and subsequent cardiac remodeling. Cardiac remodeling is the process of structural and functional changes that lead to a progressively dilated LV and impaired contractile function. Early phase ventricular remodeling occurs within the first few days after infarction and involves regional thinning and expansion of the infarct zone and ventricular dilation (Hutchins & Bulkley, 1978; Pfeffer et al., 1991). Elevated mechanical stress on the heart, together with local paracrine/autocrine factors and activation of systemic neurohormonal reflexes, can lead to late phase remodeling characterized by further ventricular chamber dilation (Lindpaintner et al., 1993; Meredith et al., 1993; Roberts et al., 1983). Excessive LV remodeling and dilation is a major determinant of whether patients will go on to manifest heart failure and is one of the strongest predictors of mortality after MI (Cohn et al., 1984; Kostuk et al., 1973).

The decrease in cardiac function and local mechanical/biochemical changes within the myocardium after MI trigger the activation of neurohormonal systems including the sympathetic nervous system, the renin‐angiotensin aldosterone system, as well as release of natriuretic peptides from the heart. In the short term, this can help to maintain cardiac output and perfusion of critical organs after MI. However, chronic activation of these neurohormonal systems has been shown to cause adverse ventricular remodeling and the progression to heart failure (Sutton & Sharpe, 2000). Therapies targeting these systems have become the cornerstone of treatment after MI–resulting in a reduction of LV volume and mass, increased ejection fraction, and decreased mortality (Chatterjee et al., 2013; Pfeffer, 1998). While the efficacy of neurohormonal modulation is proven, few new therapies have come forth within the past couple of decades.

Recent studies in animal models of heart failure suggest a new approach. Rather than targeting the efferent limbs of these reflexes, inhibition of the sensory (afferent) pathways that trigger these neurohormonal pathways has shown beneficial effects on cardiac remodeling and cardiovascular function (Schultz et al., 2013; Wang, Wang, et al., 2014; Yadav et al., 2026). Most of this work has focused on the sensory neurons that innervate the heart, and particularly those cardiac afferents that follow the sympathetic nerve pathways back to their cell bodies located in the dorsal root ganglia (termed “cardiac sympathetic afferents”). Besides signaling pain during myocardial ischemia or infarction, cardiac sympathetic afferents also trigger sympathoexcitation (Malliani et al., 1969; Minisi & Thames, 1991). This 'cardiac sympathetic afferent reflex' is enhanced in heart failure and is a major contributor to sympathoexcitation in heart failure (Chen et al., 2015; Wang & Zucker, 1996). In a rat model of heart failure after MI, intrapericardial injection of resiniferotoxin (transient receptor potential vanilloid 1 [TRPV1] receptor agonist) to ablate cardiac sympathetic afferents prevented sympathoexcitation, improved baroreflex sensitivity, and also reduced cardiac dilation and improved cardiac diastolic function (Wang et al., 2017; Wang, Wang, et al., 2014). Other studies have evaluated the potential role of TRPV1, a channel expressed in cardiac afferents, but found that global knockouts of TRPV1 actually worsened inflammation, fibrosis, and deleterious remodeling after MI (Huang et al., 2009, 2010).

As an organ with high metabolic activity, the heart is susceptible to rapid drops in pH during myocardial ischemia (Yan & Kleber, 1992), and acidosis is a potent activator of cardiac sympathetic afferent fibers in vitro (Pan et al., 1999; Uchida & Murao, 1975). In isolated labeled cardiac sympathetic afferent neurons we found that acid solution evoked large depolarizing currents, and their current properties were consistent with acid‐sensing ion channels (ASICs) (Benson et al., 1999; Sutherland et al., 2001). ASICs belong to the DEG/ENaC family of ion channels that include four genes (*ASIC1*, ‐*2*, ‐*3*, and *‐4*) encoding 6 subunits (ASIC1a, ‐1b, ‐2a, ‐2b, ‐3, ‐4) in rodents. Functional ASIC channels consist of a complex of three subunits that can form homomultimeric channels with three of the same subunits or heterotrimers composed of two or more subunits (Jasti et al., 2007; Wemmie et al., 2006). Besides being activated by interstitial acidosis, ASICs are activated or potentiated by other metabolites that are released by ischemic tissue including lactate, arachidonic acid, and ATP–suggesting that ASICs may serve as metabolic and pain sensors during stress conditions such as ischemia (Allen & Attwell, 2002; Birdsong et al., 2010; Immke & McCleskey, 2001). We have subsequently shown that the composition of ASIC channels in murine cardiac sympathetic afferent neurons is heteromeric composed of ASIC2a and ASIC3 subunits, with ASIC3 being responsible for its precise pH sensitivity in the ranges that occur during myocardial ischemia (Hattori et al., 2009; Yagi et al., 2006).

Given that (1) cardiac sympathetic afferents are important triggers of sympathoexcitation and deleterious cardiac remodeling after MI, (2) ASIC3 is highly expressed and is a sensitive pH sensor within cardiac sympathetic afferents, and (3) ASIC3 is generally coexpressed in the same set of sensory neurons that express TRPV1 (the target of resiniferotoxin) (Leffler et al., 2006), we tested if genetic deletion of ASIC3 would alter cardiac remodeling and autonomic function after MI. To test this, cardiac morphology was evaluated using echocardiography before and after the induction of an MI via ligation of the left anterior descending artery in wild type and ASIC3^−/−^ mice. Unlike other ASIC subunits, which are expressed in mouse brain, ASIC3 is only functionally expressed in peripheral sensory neurons (Lingueglia et al., 1997; Price et al., 2001). Thus, a global ASIC3 knockout serves as a good model to test the role of sensory neurons in cardiac remodeling after MI.

## 2 MATERIALS AND METHODS

### 2.1 Ethical approval

All experimental procedures and protocols were approved by the Institutional Animal Care and Use Committee of the University of Iowa (Protocol no. 2051770) and conformed to the national guidelines set by the Association for Assessment and Accreditation of the Laboratory Animal Care.

### 2.2 Animals

ASIC3^−/−^ mice were generated (Price et al., 2001) and bred on a C57Bl/6J background and were compared to wild‐type (WT) C57Bl/6J mice (Jackson Laboratories). These mice were subsequently backcrossed for 10 generations onto a C57Bl/6J background to generate a congenic line. Following the initial development of the strain, the ASIC3^−/−^ mouse line was backcrossed every 10 generations to avoid genetic drift. All mice were housed at the University of Iowa in a temperature‐controlled room (22C°) with a 12‐h light/dark cycle with ad libitum access to both water and standard mouse chow (inotiv, Irradiated Uniprim Diet, TD.06596). Both male and female mice were used in all groups. Since there were no sex differences in cardiac remodeling after MI, we analyzed them together (Table S1). Mice were euthanized with CO_2_ per AVMA guidelines.

### 2.3 Echocardiography

Cardiac function was measured at baseline by echocardiography in 7‐week‐old, lightly‐sedated mice (midazolam 0.1 mg subcutaneous injection) using a Vevo 2100 instrument (Visualsonics, Toronto, Canada) equipped with a 30‐MHz probe as previously described (Zhang et al., 2020). Briefly, the anterior chest hair was shaved and warmed gel was applied. The mouse was scruffed and held in the left lateral position. Images of the short and long axis were obtained with a frame rate of ~180–250 Hz. All image analysis was performed using Vevo 2100 analysis software version 5.7.1, by an investigator blinded to genotype. Endocardial and epicardial borders were traced on the short axis view at end‐diastole and end‐systole. LV length was measured from endocardial and epicardial borders to the LV outflow tract in end‐diastole and end‐systole. The biplane area‐length method was performed to calculate LV volumes and mass. We previously found a tight correlation between echocardiographic and isolated and weighed LV mass (Hill et al., 2000). 48 h after surgery echocardiograms were performed without sedation to evaluate the ligation‐induced myocardial injury. Ischemic zone (IZ) fraction was reported as the non‐contractile fraction of the LV by tracing the endocardial silhouette in the short axis, which has been shown to correlate well with histologically measured infarct size (Dann et al., 2022; Lei et al., 2004). Three weeks post‐surgery another echocardiogram was done without sedation to evaluate cardiac remodeling. Remodeling data are presented as the change between 48 h and 3 weeks to represent the late remodeling that occurs as a result of the initial injury. Previous data in mice after coronary ligation show that most of the cardiac remodeling occurs by 2.5 weeks and remains largely unchanged through 6 weeks post‐MI (Gao et al., 2000).

### 2.4 MI/sham surgery

One week following baseline echocardiograms, ASIC3^−/−^ and WT mice (8 weeks of age) underwent MI or sham surgery as previously described (Chen et al., 2012). Animals were anesthetized with isoflurane, prepared, and ventilated. Hearts were accessed via a thoracotomy and infarcted by placing a permanent ligature on the mid‐left anterior descending coronary artery using aseptic technique. The ribcages were then closed with the air evacuated and the incision closed. The sham procedure followed the same protocol while omitting the artery ligation. Mice were then monitored continuously until awake, and analgesia (0.1 mg/kg buprenorphine) was administered immediately and daily for 2 days postoperatively. Mice were returned to their home cages with sex‐matched litter mates and monitored two times daily for 5 days. Survival rates following MI at 3 weeks were not different between the genotypes (89.3% for WT and 83.3% for ASIC3^−/−^ mice) with most deaths occurring in the first week following MI (data not included). Mice that underwent MI surgery but did not develop an MI according to echocardiography were excluded.

### 2.5 Radiotelemetry recordings of hemodynamics

One week after MI/Sham surgery, radio telemeters were implanted in a cohort of mice as previously described (Mason et al., 2020). Briefly, under isoflurane anesthesia a pressure‐sensing implantable mouse transmitter (TA11PA‐C10, DSI) was inserted in the left common carotid artery and advanced into the aorta through a midline incision. The body of the transmitter was tunneled subcutaneously to the left flank, and the incision closed. Following surgery, the analgesic flunixin meglumine (0.05 μg/g) was injected subcutaneously. The mice were individually housed and monitored daily. After the 3‐week echocardiogram, cages were transferred to the telemeter room and allowed to acclimate for 2 days before turning on telemeters. Heart rate and blood pressure were continuously recorded for 48 h. Following these recordings, cardiovagal and cardiac sympathetic tone were measured from HR responses to methylatropine (1 mg/kg, intraperitoneal [i.p.]) and propranolol (1 mg/kg, i.p.) respectively. Autonomic modulation of cardiac function was also assessed via heart rate variability using both the root mean square of successive differences (RMSSD, a measure of vagal modulation) and power spectral analysis from the 48‐h recordings before drug injections (Stauss, 2007). For spectral analysis, the low frequency (LF) band was set at 0.2–0.8 Hz and the high frequency (HF) band was set at 0.8–5.0 Hz (Baudrie et al., 2007). Baroreceptor‐heart rate reflex sensitivity was calculated using the sequence technique as previously described (Stauss et al., 1997). Briefly, sequences of three or more consecutive heart beats where both arterial blood pressure and interbeat intervals simultaneously increased or decreased were detected. Linear regressions were calculated for these sequences.

### 2.6 Sympathetic nerve recording

In a sub cohort of mice following 3‐week echocardiograms, regional sympathetic nerve activity (SNA) was measured using direct multifiber recording in anesthetized mice as previously described (Rouabhi et al., 2021). Mice were anesthetized with i.p. injection of ketamine (91 mg/kg) and xylazine (9.1 mg/kg). Each mouse was intubated (PE‐50) to allow for spontaneous respiration of oxygen‐enriched room air. Body temperature, measured with a rectal probe, was kept constant at 37.5°C by using a surgical heat lamp and a metal heat platform. The left jugular vein was cannulated with a micro‐renathane tubing (MRE‐40) to sustain the level of anesthesia throughout the 4‐h protocol with α‐chloralose (initial dose: 12 mg/kg; sustaining dose of 6 mg/kg/h) and for drug treatments. Finally, the left carotid artery was cannulated with a tapered micro‐renathane tubing (MRE‐40) for continuous measurement of arterial pressure and heart rate.

A dissecting microscope was employed: the nerves subserving the left kidney (RSNA) and visceral organs (splanchnic SNA) were identified, carefully dissected free, and placed on a bipolar 36‐gauge platinum‐iridium electrode (A‐M Systems). When the optimum recording of SNA was obtained from each nerve, the electrode was covered with silicone gel (Kwik‐Sil; World Precision Instruments Inc). The electrode was attached to a high‐impedance probe (HIP‐511, Grass Instruments), and the nerve signal was amplified 10^5^ times with a Grass P5 AC pre‐amplifier. The amplified nerve signal was bandpass filtered between 100 Hz and 1000 Hz and was then routed to a speaker system and to an oscilloscope (model 54,501 A, Hewlett–Packard) to monitor the audio and visual quality of the sympathetic nerve recordings. The amplified, filtered nerve signal was also directed to a MacLab analogue‐digital converter (Model 8 S, AD Instruments Castle Hill) containing software (MacLab Chart Pro; Version 7.0) that uses a cursor to analyze the number of spikes/s that exceeds the background noise threshold.

Arterial baroreflex control of renal SNA was tested by intravenous infusion of sodium nitroprusside (at doses of 0.05, 0.1, 0.5, and 1 μg) followed by phenylephrine (at doses of 0.05, 0.1, 0.5, and 1 μg), which decreases and increases arterial pressure, respectively. To ensure that background electrical noise was excluded from sympathetic measurements, post‐mortem background activity was subtracted from all SNA. Multi‐unit activity was analyzed, and sympathetic discharges were identified and quantified using established conduction‐ and pattern‐based criteria.

### 2.7 Quantitative PCR

Whole hearts were extracted and homogenized in TRIzol™ Reagent (Invitrogen, 15596026). RNA was extracted following the manufacturer's protocol. RNA was quantified using a Nano drop spectrophotometer. Reverse transcription was done using 1 μg of RNA with the High‐capacity cDNA Reverse Transcription Kit (Applied Biosystems™, 4368814). Quantitative PCR was done using the *Power* Sybr™ Green PCR Master Mix (ThermoFisher, 4367659) and read on the Applied Biosystems QuantStudio 7 Flex PCR instrument. Data were analyzed using the 2^−ΔΔCt^ method and the respective Sham group as the calibrator. Primer sequences for the target genes are listed in Table 1.

**TABLE 1:** qPCR primer sequences.

Gene	PrimerBank reference number	Forward primer sequence (5′‐3′)	Reverse primer sequence (5′‐3′)
18S (Wang, Leinwand, & Anseth, 2014)		GCCGCTAGAGGTGAAATTCTT	CTTTCGCTCTGGTCCGTCTT
α‐SMA (Mao et al., 2015)		CCCAGACATCAGGGAGTAATGG	TCTATCGGATACTTCAGCGTCA
Myh6	255918223c1	GCCCAGTACCTCCGAAAGTC	ATCAGGCACGAAGCACTCC
Myh7	118131045c1	CCTGCGGAAGTCTGAGAAGG	CTCGGGACACGATCTTGGC
Nppa	142378163c1	GTGCGGTGTCCAACACAGAT	TCCAATCCTGTCAATCCTACCC
Nppb	161621278c1	GAGGTCACTCCTATCCTCTGG	GCCATTTCCTCCGACTTTTCTC
Col1a2	111120328c1	TCGTGCCTAGCAACATGCC	TTTGTCAGAATACTGAGCAGCAA
Col3a1	20380522a1	CTGTAACATGGAAACTGGGGAAA	CCATAGCTGAACTGAAAACCACC

*Note*: Sequences were taken from the Harvard Medical School PrimerBank unless otherwise indicated.

Abbreviations: Col1a2, type 1 collagen; Col3a1, type 3 collagen; Myh6, α‐myosin heavy chain; Myh7, β‐myosin heavy chain; Nppa, atrial natriuretic peptide; Nppb, brain natriuretic peptide; SMA, smooth muscle actin.

### 2.8 Statistics

GraphPad Prism (10.4.1) was used to analyze statistical data. Bar graphs represent means ± standard error of the mean (SEM). Continuous recording data is presented as mean ± standard deviation (SD). Three‐way ANOVA, two‐way ANOVA, unpaired *t*‐test, simple linear regression, and chi square were used as appropriate to analyze results. If significant differences were observed, the indicated post hoc tests as described in the figure legends were performed. Significance was reported at *p* < 0.05.

## 3 RESULTS

### 3.1 ASIC3 ^−/−^ mice have improved stroke volume and undergo less LV dilation relative to infarct size after MI than WT mice

**FIGURE 1:**
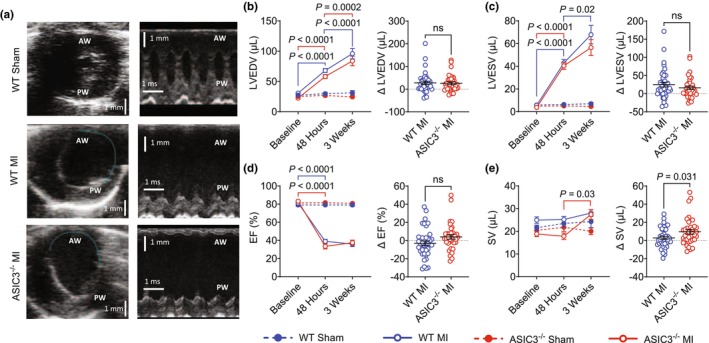
Echocardiographic morphometric data measured before and after (48 h and 3 weeks) myocardial infarction (MI) or sham surgery in wild type (WT) and ASIC3^−/−^ mice. (a) Representative images recorded at 3 weeks after MI or sham surgery. Left column: Short‐axis echocardiograms at end‐systole. Teal: endocardial silhouette of akinetic myocardium. Right column: M‐mode echocardiograms. Anterior left ventricular wall (AW), posterior left ventricular wall (PW). (b) Left ventricular end diastolic volume (LVEDV): Three‐way ANOVA revealed a significant effect of time (*F*_(2,230)_ = 59.9, *p* ≤ 0.0001), surgery (*F*_(1,115)_ = 82.63, *p* ≤ 0.0001) and a significant time × surgery interaction (*F*_(1,115)_ = 54.02, *p* ≤ 0.0001). Tukey post‐hoc test found significant differences for WT MI between baseline and 48 h and 48 h and 3 weeks and for ASIC3^−/−^ MI between baseline and 48 and 48 h and 3 weeks. (c) Left Ventricular End Systolic Volume (LVESV): Three‐way ANOVA revealed a significant effect of time (*F*_(2,230)_ = 60.59, *p* ≤ 0.0001), surgery (*F*_(1,115)_ = 97.73, *p* ≤ 0.0001) and a significant time × surgery interaction (*F*_(1,115)_ = 58.87, *p* ≤ 0.0001). Tukey post‐hoc test found significant differences for WT MI between baseline and 48 and 48 h and 3 weeks and for ASIC3^−/−^ MI between baseline and 48 h. (d) ejection fraction (EF): Three‐way ANOVA revealed a significant effect of time (*F*_(2,230)_ = 201.5, *p* ≤ 0.0001), surgery (*F*_(1,115)_ = 345.5, *p* ≤ 0.0001) and a significant time × surgery interaction (*F*_(1,115)_ = 200.9, *p* ≤ 0.0001). Tukey post‐hoc test found significant differences for WT MI between baseline and 48 h and for ASIC3^−/−^ MI between baseline and 48 h. (e) Stroke Volume (SV): Three‐way ANOVA revealed a significant effect of time (*F*_(2,230)_ = 6.01, *p* = 0.003), genotype (*F*_(1,115)_ = 9.29, *p* = 0.003) and a significant time × surgery interaction (*F*_(1,115)_ = 5.14, *p* = 0.007). Tukey post‐hoc test found a significant difference for ASIC3^−/−^ MI between 48 h and 3 weeks. Unpaired Student's *t*‐tests revealed a significant difference between MI groups for SV. Values are reported as mean ± SEM. [Nonsignificant (ns), WT Sham: *N* = 21, WT MI: *N* = 39, ASIC3^−/−^ Sham: *N* = 24, ASIC3^−/−^ MI: *N* = 35].

To test if ASIC3 contributes to cardiac remodeling after MI, baseline echocardiograms were performed prior to MI/Sham surgery and then repeated at 48 h post‐op to confirm the presence and size of injury as measured by the ischemic zone (IZ), or akinetic, fraction of the LV. Three weeks after surgery a third echocardiogram was performed to quantify cardiac remodeling. Since there were no sex differences in cardiac remodeling after MI, we analyzed male and female mice together (Table S1). Figure 1a shows representative short‐axis echocardiographic images in systole (*left column*) and m‐mode images (*right* column) for a WT Sham (*top*), WT MI (*middle*), and ASIC3^−/−^ MI (*bottom*) heart at 3 weeks post‐MI. Compared to baseline, echocardiograms at 48 h post‐MI revealed that both WT and ASIC3^−/−^ MI mice underwent a significant increase in LV end‐diastolic volume (LVEDV) (Figure 1b: *left*) and LV end‐systolic volume (LVESV) (Figure 1c: left), a significant decrease in LV ejection fraction (EF) (Figure 1d: left), and no change in stroke volume (SV) (Figure 1e: left). While early remodeling can occur within the first 48 h after MI, most late remodeling happens between 48 h and 3 weeks after MI in mice (Gao et al., 2000; Protti et al., 2012). Both WT MI and ASIC3^−/−^ MI mice underwent further increases in LVEDV at 3 weeks compared to 48 h (Figure 1b: left). WT MI mice also had a further significant increase in LVESV between 48 h and 3 weeks; however, the change in LVESV of ASIC3^−/−^ MI mice was not significant (Figure 1c: left). Neither the WT nor the ASIC3^−/−^ MI mice saw changes in EF between 48 h and 3 weeks (Fig D: left). In addition, ASIC3^−/−^ MI mice had a significant increase in stroke volume between 48 h and 3 weeks, whereas WT mice did not (Figure 1e: left). To further quantify LV remodeling, we compared the change in each parameter between 48 h and 3 weeks after MI between MI groups. Whereas there were no significant differences between WT and ASIC3^−/−^ mice in ΔLVEDV, ΔLVESV, and ΔLVEF (Figure 1b–d: right), ASIC3^−/−^ MI mice increased their LV stroke volume between 48 h and 3 weeks more than the WT MI mice (Figure 1e; right).

**FIGURE 2:**
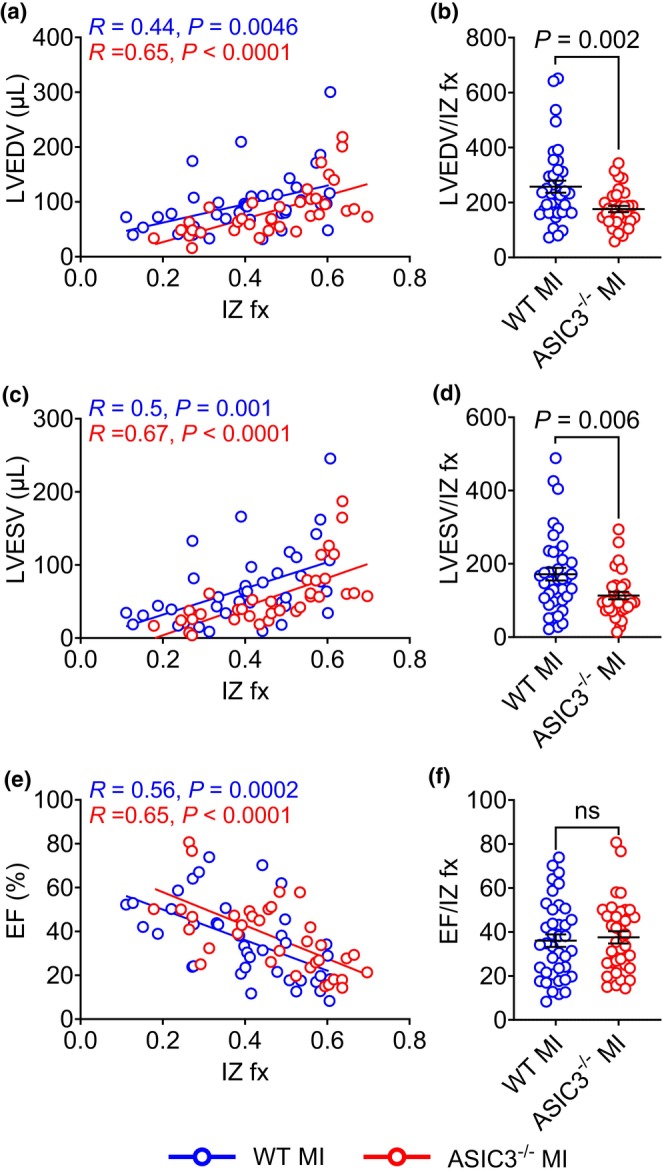
Effect of myocardial infarction (MI) size on cardiac remodeling after MI. (a) Correlation of left ventricular end diastolic volume (LVEDV) to IZ fx. Simple linear regression testing revealed significant correlations for both WT MI and ASIC3^−/−^ MI, as well as a significant difference in the y‐intercepts of the groups (*F*_(1,71)_ = 5.89, *p* = 0.018); lines are best‐fit regressions. (b) LVEDV normalized to IZ fx. Unpaired *t*‐test revealed a significant difference between groups. (c) Correlation of left ventricular end systolic volume (LVESV) to IZ fx. Simple linear regression testing revealed significant correlations for both WT MI and ASIC3^−/−^ MI, as well as a significant difference in the *y*‐intercepts of the groups (*F*_(1,71)_ = 6.96, *p* = 0.01). (d) LVESV normalized to IZ fx. Unpaired *t*‐test revealed a significant difference between groups. (e) Correlation of left ventricular ejection fraction (LVEF) to IZ fx. Simple Linear Regression testing revealed significant correlations for both WT MI and ASIC3^−/−^ MI, as well as a trend towards a significant difference in the y‐intercepts of the groups (*F*_(1,71)_ = 3.82, *p* = 0.055). (F) LVEF normalized to IZ fx. Values are reported as mean ± SEM. (ns, nonsignificant; WT MI: *N* = 39, ASIC3^−/−^ MI: *N* = 35).

Left anterior descending artery ligation generates infarctions of variable size in mice, which can lead to a variability in the degree of remodeling. To investigate the effect of ischemic injury on the cardiac remodeling, we correlated the 3‐week echo data to IZ fraction (see Figure 1a). As expected, both the WT and ASIC3^−/−^ mice saw a positive correlation between LV volumes at 3 weeks post‐MI and IZ fraction. When fitted with a simple linear regression, the slopes of the LVEDV (Figure 2a) and LVESV (Figure 2c) were not different from each other. However, the y‐intercepts of the LVEDV and LVESV for the ASIC3^−/−^ mice were significantly lower than the WT mice. Thus, ASIC3^−/−^ mice had less LV dilation for any given IZ fraction across the wide spectrum of LV injury. Correlation linear regression fits of LVEF to infarction size show that the slopes of the lines were not different and that the y‐intercept of the ASIC3^−/−^ MI mice trended towards greater LVEF compared to WT mice after MI (*p* = 0.055; Figure 2e). Additionally, we normalized the 3‐week echocardiogram data with the IZ fraction (Figure 2b,d,f) and found LVEDV and LVESV to be lower in ASIC3^−/−^ mice compared to WT mice. In summary, these results indicate that ASIC3^−/−^ mice undergo less LV dilation and have improved stroke volume compared to WT mice after MI.

### 3.2 LV mass and wall thickness increases in ASIC3 ^−/−^ but not WT mice after MI

**FIGURE 3:**
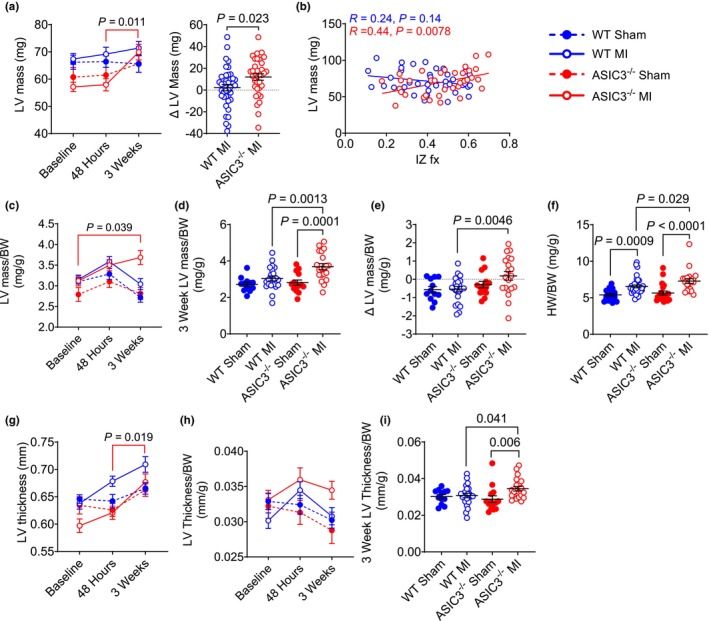
Change in left ventricular (LV) mass after myocardial infarction (MI). (a) Left: LV mass measured from echocardiograms plotted over time in WT (blue) and ASIC3^−/−^ (red) mice after sham (closed circles) or MI (open circles) surgery. Three‐way ANOVA revealed a significant effect of time (*F*_(2,230)_ = 6.93, *p* = 0.001), genotype (*F*_(1,115)_ = 7.51, *p* = 0.007) and a significant time × genotype interaction (*F*_(2,230)_ = 3.88, *p* = 0.022). Tukey post‐hoc test found significant differences for ASIC3^−/−^ MI between 48 h and 3 weeks. Right: The change in LV mass from 48 h post‐MI to 3 weeks post‐MI in WT and ASIC3^−/−^ mice after sham or MI surgery. Unpaired *t*‐test revealed a significant difference between MI groups. (b) Correlation between 3‐week LV mass and ischemic zone fraction (IZ fx) in WT and ASIC3^−/−^ mice. Simple Linear Regression testing revealed a significant correlation for ASIC3^−/−^ MI but not WT MI, and a significant difference in the slopes of the groups (*F*_(1,70)_ = 9.84, *p* = 0.0025); lines are best fit regressions (WT Sham: *N* = 21; WT MI: *N* = 39, ASIC3^−/−^ Sham: *N* = 24; ASIC3^−/−^ MI: *N* = 35). (c) LV mass normalized to body weight (BW) at indicated intervals in a subset of mice in which body weight was obtained at each time point. Three‐way ANOVA revealed a significant effect of time (*F*_(2,128)_ = 7.46, *p* = 0.0009), surgery (*F*_(1,64)_ = 17.42, *p* ≤ 0.0001) and a significant time × genotype interaction (*F*_(2,128)_ = 5.15, *p* = 0.007). Tukey post‐hoc test found a significant difference for ASIC3^−/−^ MI between baseline and 3 weeks. (d) LV mass at 3 weeks normalized to body weight for data in c. Two‐way ANOVA revealed a significant effect of surgery (*F*_(1,64)_ = 14.9, *p* ≤ 0.0001) and genotype (*F*_(1,64)_ = 5.69, *p* = 0.020). Fisher's LSD post‐hoc test found significant differences between WT MI and ASIC3^−/−^ MI and ASIC3^−/−^ Sham and ASIC3^−/−^ MI. (e) Change in LV Mass normalized to BW between 48 h and 3 weeks post‐MI for data in c. Two‐way ANOVA revealed a significant effect of genotype (*F*_(1,64)_ = 6.31, *p* = 0.015). Fisher's LSD post‐hoc test found a significant difference between ASIC3^−/−^ MI and WT MI. (WT Sham: *N* = 12; WT MI: *N* = 22, ASIC3^−/−^ Sham: *N* = 14, ASIC3^−/−^ MI: *N* = 20). (f) Heart weight (HW) normalized to BW taken at 3 weeks post‐MI. Two‐way ANOVA revealed a significant surgery effect (*F*_(1,91)_ = 30.82, *p* < 0.0001), with a trend towards a significant genotype effect (*F*_(1,91)_ = 3.86, *p* = 0.053). Fisher's LSD post‐hoc test found a significant difference between WT Sham and WT MI, ASIC3^−/−^ Sham and ASIC3^−/−^ MI, and WT MI and ASIC3^−/−^ MI. (WT Sham: *N* = 19; WT MI: *N* = 35, ASIC3^−/−^ Sham: *N* = 20, ASIC3^−/−^ MI: *N* = 21). (g) LV thickness at indicated intervals. Three‐way ANOVA revealed a significant effect of time (*F*_(2,226)_ = 17.73, *p* < 0.0001), genotype (*F*_(1,113)_ = 10.22, *p* = 0.002), a significant time × surgery interaction (*F*_(2,226)_ = 4.79, *p* = 0.009), and a significant genotype × surgery interaction (*F*_(1,113)_ = 3.97, *p* = 0.049). Tukey post‐hoc test found significant differences for ASIC3^−/−^ MI between 48 h and 3 weeks. (WT Sham: *N* = 21; WT MI: *N* = 39, ASIC3^−/−^ Sham: *N* = 23; ASIC3^−/−^ MI: *N* = 34) (h) LV thickness normalized to body weight (BW) at indicated intervals in a subset of mice in which body weight was obtained at each time point. (WT Sham: *N* = 21; WT MI: *N* = 39, ASIC3^−/−^ Sham: *N* = 24; ASIC3^−/−^ MI: *N* = 35). (i) LV thickness at 3 weeks for data in h. Two‐way ANOVA revealed a significant effect of surgery (*F*_(1,64)_ = 4.75, *p* = 0.033). Fisher's LSD post‐hoc test found significant differences between WT MI and ASIC3^−/−^ MI and ASIC3^−/−^ Sham and ASIC3^−/−^ MI. (WT Sham: *N* = 12; WT MI: *N* = 22, ASIC3^−/−^ Sham: *N* = 14; ASIC3^−/−^ MI: *N* = 20). Values are reported as mean ± SEM. ns, nonsignificant.

As a measure of hypertrophy, we measured LV mass via echocardiography at baseline and at 48 h and 3 weeks after MI. Between 48 h and 3 weeks, ASIC3^−/−^ MI mice increased their LV mass significantly more than WT MI mice and were the only group to undergo an increase in mass between 48 h and 3 weeks (Figure 3a). Simple linear regression of the correlation between LV mass at 3 weeks post‐MI and IZ fraction showed a positive correlation in the ASIC3^−/−^ MI but not in WT MI mice (Figure 3b). This implies that ASIC3^−/−^ MI mice underwent increasing LV hypertrophy per increase in IZ fraction, whereas this did not occur in WT MI mice. To validate that the increase in mass wasn't due to differences in animal size, we normalized these results to body weight in a subset of animals for which body weights at all three time points were recorded (Figure 3c, Table S2). ASIC3^−/−^ MI mice had a significant increase in LV mass when normalized to body weight between baseline and 3 weeks, whereas no other groups saw an increase (Figure 3c). When normalized to body weight, ASIC3^−/−^ MI mice had greater LV mass at 3 weeks compared to ASIC3^−/−^ Sham and WT MI mice (Figure 3d), and the change in normalized LV mass at 3 weeks compared to 48 h was also greater in ASIC3^−/−^ mice than in WT (Figure 3e). Hearts were then collected and weighed after the 3‐week echocardiogram. Heart weight (HW) to BW ratio was significantly higher in both MI groups compared to their sham counterparts, and ASIC3^−/−^ mice had a larger HW/BW than WT mice after MI (Figure 3f). Additionally, LV thickness was measured via echocardiography on the short axis view at end‐diastole as the widest part of the septal wall, and between 48 h and 3 weeks, ASIC3^−/−^ MI mice were the only group to significantly increase LV thickness (Figure 3g). When normalized to body weight in a subset of animals (Figure 3h), the ASIC3^−/−^ MI group had greater LV thickness at 3 weeks than ASIC3^−/−^ Sham and WT MI mice (Figure 3i).

### 3.3 Altered neurohormonal responses in ASIC3 ^−/−^ mice compared to WT mice after MI

**FIGURE 4:**
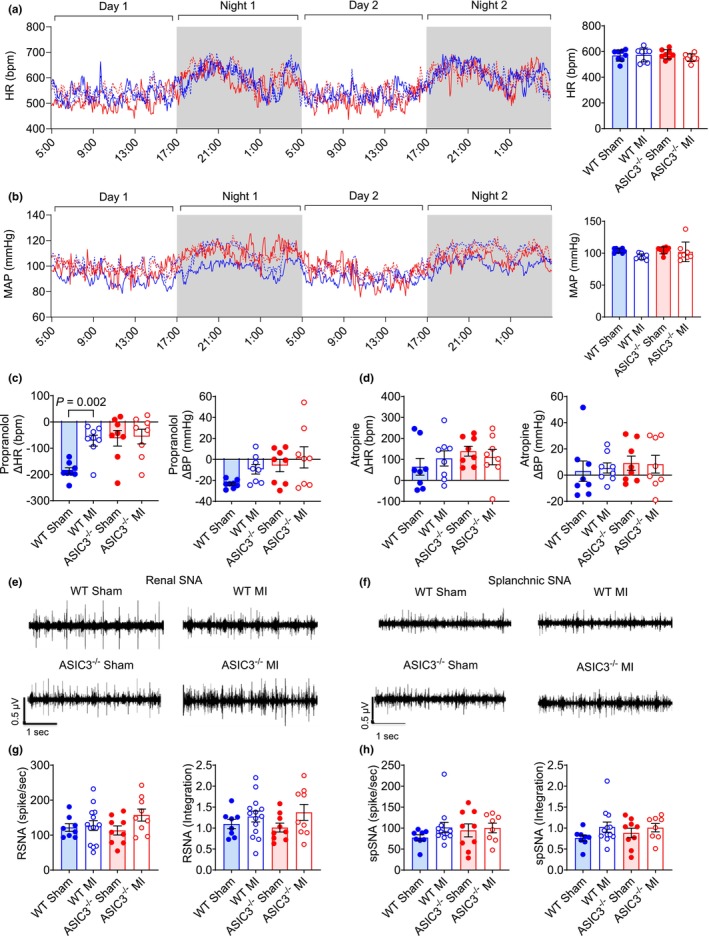
Measure of autonomic nervous system tone. (a) Average heart rate of radio‐telemeter implanted wild‐type (WT; blue) and ASIC3^−/−^ (red) mice over 2 days. Traces of heart rate over the 2‐day period (*left*) and average of the 2 days (*right*). Two‐way ANOVA of HR data with Fisher's LSD post‐hoc adjustment showed no significant effects. (b) Mean arterial pressure (MAP) of radio‐telemeter implanted WT and ASIC3^−/−^ mice over 2 days. Traces of BP over the 2‐day period (*left*) and average of the 2 days (*right*). Two‐way ANOVA with Fisher's LSD post‐hoc adjustment of MAP data showed no significant effects. A significant difference was observed between WT Sham and WT MI. Values are reported as mean ± SD. (c) Change in heart rate (HR, *left*) and blood pressure (BP, *right*) after injection of 0.1 mg/kg propranolol. Two‐way ANOVA revealed a significant effect of surgery (*F*_(1,28)_ = 4.68, *p* = 0.039). Fisher's LSD post‐hoc test found significant differences between WT Sham and WT MI, WT Sham and ASIC3^−/−^ Sham, and WT Sham and ASIC3^−/−^ MI. (d) Change in HR (*left*) and BP (*right*) after injection of 0.1 mg/kg atropine. Two‐way ANOVA with Fisher's LSD post‐hoc test found no significant effects (*N* = 8 for each group). (e) Representative basal renal SNA traces. (f) Representative basal splanchnic SNA traces. (g) Renal sympathetic nerve activity (RSNA). Two‐way ANOVA with a Fisher's LSD post‐hoc adjustment found no significant effects. (WT Sham: *N* = 8; WT MI: *N* = 14, ASIC3^−/−^ Sham: *N* = 9, ASIC3^−/−^ MI: *N* = 9). (h) Splanchnic sympathetic nerve activity (spSNA). Two‐way ANOVA with Fisher's LSD post‐hoc test found no significant effects. Values are reported as mean ± SEM (WT Sham: *N* = 8; WT MI: *N* = 12, ASIC3^−/−^ Sham: *N* = 9, ASIC3^−/−^ MI: *N* = 8).

To explore the underlying mechanisms that lead to altered cardiac remodeling in ASIC3^−/−^ mice compared to WT mice after MI, we first recorded hemodynamics continuously for 2 days in conscious mice in their home cage using radiotelemetry (Table S3). The average heart rate (HR) and the mean arterial blood pressure (MAP) did not differ between any of the groups over the 48‐h period (Figure 4a,b). Next, we evaluated baseline parasympathetic and sympathetic autonomic tone. As a measure of cardiac sympathetic tone in conscious mice, we recorded HR and BP responses to acute injection of propranolol (β‐adrenergic receptor blocker). Propranolol decreased HR in all groups, with a significant decrease in the WT sham mice compared to WT MI mice, but no difference between MI groups (Figure 4c, *left*). BP was also decreased in the WT sham mice, but no differences were seen between the groups (Figure 4c, right). As a measure of cardiac vagal tone, acute injection of atropine (muscarinic cholinergic receptor blocker) increased HR in all groups and had a marginal effect on BP, but no differences were seen between the groups (Figure 4d). In a separate cohort of mice, we directly recorded sympathetic nerve activity (SNA) from both the renal and splanchnic nerves in anesthetized mice (Figure 4e,f, respectively). Unexpectedly, we did not see a significant increase in SNA following MI in either genotype, and no differences were observed between the genotypes in either the renal SNA activity (Figure 4g) or the splanchnic SNA (Figure 4h).

**FIGURE 5:**
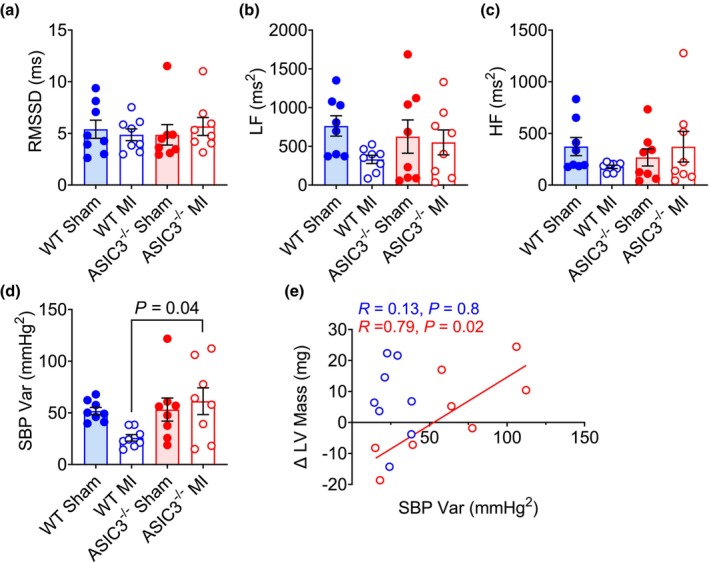
Heart rate and blood pressure variability. (a) Root mean square of successive differences (RMSSD). Two‐way ANOVA with Fisher's LSD post‐hoc test found no significant effects. High frequency (HF) range (b) and low frequency (LF) range (c) from power spectra analysis. Two‐way ANOVA with Fisher's LSD post‐hoc test found no significant effects. (d) Systolic blood pressure (SBP) variance. Two‐way ANOVA revealed a significant effect of genotype (*F*_(1,28)_ = 4.33, *p* = 0.047). Tukey post‐hoc test found a significant difference between WT MI and ASIC3^−/−^ MI. (e) Correlation between SBP variance and change in LV mass. Simple linear regression testing revealed a significant correlation for ASIC3^−/−^ MI but not WT MI. (WT MI: *N* = 8, ASIC3^−/−^ MI: *N* = 8).

As additional measures of autonomic function, we measured HR and BP variability. HRV expressed as RMSSD obtained from telemeter‐implanted, conscious mice showed no differences at 3 weeks after MI or any differences between genotypes (Figure 5a). We also evaluated the contribution of different oscillatory components of HRV via power spectral analysis and found no differences in low frequency (Figure 5b) or high frequency spectral powers (Figure 5c). On the other hand, ASIC3^−/−^ mice had a significantly higher systolic blood pressure variability (BPV) than the WT mice after MI (Figure 5d). We also found that the increase in LV mass from 48 h to 3 weeks post‐MI significantly correlated with BPV in ASIC3^−/−^ MI mice but not in WT MI mice (Figure 5e).

**FIGURE 6:**
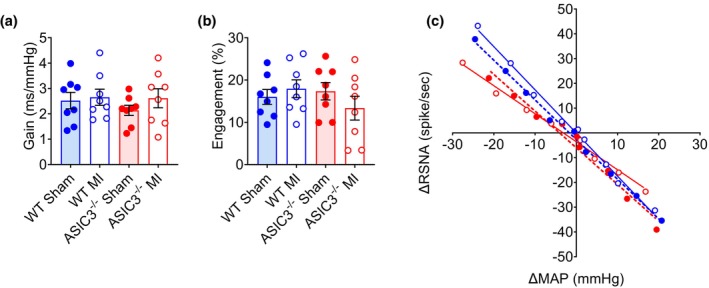
Baroreflex sensitivity. (a) Baroreflex gain and engagement (b) as measured via telemetry in conscious mice. Two‐way ANOVA with Fisher's LSD post‐hoc adjustment found no significant effects. (WT Sham: *N* = 8; WT MI: *N* = 8, ASIC3^−/−^ Sham: *N* = 8, ASIC3^−/−^ MI: *N* = 8). (c) Baroreflex sensitivity determined by the response of renal sympathetic nerve activity (RSNA) to changes in mean arterial pressure (MAP) evoked by varying doses of sodium nitroprusside and phenylephrine. Two‐way ANOVA of the slopes determined by simple linear regression revealed a significant effect of genotype (*F*_(1,325)_ = 11.25, *p* = 0.0009) and a surgery × genotype interaction (*F*_(1,325)_ = 5.05, *p* = 0.025). Tukey post‐hoc test found significant differences between WT Sham and ASIC3^−/−^ MI (*p* = 0.023) and WT MI and ASIC3^−/−^ MI (*p* = 0.0003). (WT Sham: *N* = 8; WT MI: *N* = 14, ASIC3^−/−^ Sham: *N* = 9, ASIC3^−/−^ MI: *N* = 9).

An increase in BPV is often associated with or driven by a decrease in baroreflex sensitivity. Baroreflex sensitivity can be used as a measure of autonomic responsiveness and is known to be diminished following myocardial infarction (Salah et al., 2025). Using our baseline telemetry data, we utilized spontaneous fluctuation in BP and HR to calculate the baroreflex gain (Figure 6a) and engagement (Figure 6b) using the sequence technique in conscious, free‐ranging mice. We did not see a diminishment in gain or engagement after MI in either group or any differences between genotypes. In addition to baroreceptor‐heart rate reflex sensitivity, we also evaluated baroreceptor‐renal sympathetic nerve activity (RSNA) reflex sensitivity in anesthetized mice by measuring the response of RSNA to changes in MAP evoked by infusion of varying doses of the vasodilator sodium nitroprusside followed by varying doses of the vasoconstrictor phenylephrine. ASIC3^−/−^ MI mice had reduced baroreceptor‐RSNA reflex sensitivity compared to WT MI mice (Figure 6c).

**FIGURE 7:**
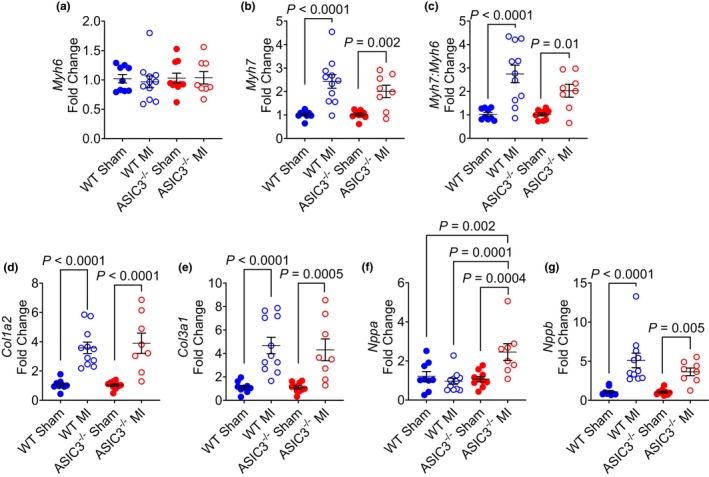
Quantitative PCR of whole mouse hearts. (a) α‐myosin heavy chain (*Myh6*). (b) β‐myosin heavy chain (*Myh7*). Two‐way ANOVA with Fisher's LSD post‐hoc adjustment revealed a significant effect of surgery (*F*_(1,34)_ = 33.71, *p* < 0.0001), with significant differences between WT Sham and WT MI, as well as ASIC3^−/−^ Sham and ASIC3^−/−^ MI. (c) Ratio of β‐myosin heavy chain to α‐myosin heavy chain (*Myh7:Myh6*). Two‐way ANOVA with Fisher's LSD post‐hoc adjustment revealed a significant effect of surgery (*F*_(1,34)_ = 28.97, *p* < 0.0001), and significant differences between WT Sham and WT MI, and ASIC3^−/−^ Sham and ASIC3^−/−^ MI. (d) Collagen type 1 (*Col1a2*). Two‐way ANOVA with Fisher's LSD post‐hoc adjustment revealed a significant effect of surgery (*F*_(1,34)_ = 50.8, *p* < 0.0001), with significant differences between WT Sham and WT MI, and ASIC3^−/−^ Sham and ASIC3^−/−^ MI. (e) Collagen type 3 (*Col3a1*). Two‐way ANOVA with Fisher's LSD post‐hoc adjustment revealed a significant effect of surgery (*F*_(1,34)_ = 34.5, *p* < 0.0001), with significant differences between WT Sham and WT MI, and ASIC3^−/−^ Sham and ASIC3^−/−^ MI. (f) Atrial natriuretic peptide (*Nppa*). Two‐way ANOVA with Fisher's LSD post‐hoc adjustment revealed a significant effect of surgery (*F*_(1,34)_ = 5.43, *p* = 0.03), genotype (F_(1,34)_ = 7.73, *p* = 0.009), and a significant surgery × genotype interaction (*F*_(1,34)_ = 11.37, *p* = 0.002), with significant differences between ASIC3^−/−^ MI and all other groups. (g) Brain natriuretic peptide (*Nppb*). Two‐way ANOVA with Fisher's LSD post hoc adjustment revealed a significant effect of surgery (*F*_(1,34)_ = 30.8, *p* < 0.0001), with differences between WT Sham and WT MI and ASIC3^−/−^ Sham and ASIC3^−/−^ MI. Values are reported as mean ± SEM normalized to respective shams. (WT Sham: *N* = 9; WT MI: *N* = 11, ASIC3^−/−^ Sham: *N* = 10, ASIC3^−/−^ MI: *N* = 8).

To further elucidate genotype differences after MI, we performed qPCR on whole mouse hearts 3 weeks after surgery. As expected (Iismaa et al., 2018), there were no differences in the mRNA expression of alpha myosin heavy chain (*Myh6*, Figure 7a) and significant elevations in the beta myosin heavy chain (*Myh7*, Figure 7b) as well as the ratio of beta to alpha myosin heavy chains (*Myh7/Myh6*, Figure 7c) following MI in both genotypes, with no differences between the MI groups. There were also significant increases in both genotypes after MI in type 1 (*Col1a2*, Figure 7d) and type 3 (*Col3a1*, Figure 7e) collagen with no difference between the MI groups. While both MI groups had elevated expression levels of the brain natriuretic peptide (*Nppb*, Figure 7g), the ASIC3^−/−^ MI group had a marked increase in the expression of atrial natriuretic peptide (*Nppa*) compared to other groups (Figure 7f).

## 4 DISCUSSION

We tested the hypothesis that ASICs within peripheral sensory neurons may contribute to neurohormonal activation and adverse cardiac remodeling following MI. The major finding of this study is that following MI, ASIC3^−/−^ mice developed less LV dilation and increased LV hypertrophy, which may have been driven by decreased baroreflex sensitivity and subsequent elevated BPV.

### 4.1 Where and how is ASIC3 affecting cardiac remodeling after MI?

Since we studied a global knock‐out model of ASIC3 deletion, an important follow‐up question is where and how ASIC3 contributes to cardiac remodeling after MI. Whereas other ASIC subunits are expressed in both peripheral neurons and the central nervous system (CNS), ASIC3 is limited to peripheral sensory neurons in rodents (Lingueglia et al., 1997; Price et al., 2001). Thus, the effects of ASIC3 deletion on cardiac remodeling likely involve the peripheral nervous system rather than direct CNS effects. We have shown that ASIC3 is expressed and probably plays a significant functional role in several afferent pathways, including cardiac afferents and within the carotid body (Sutherland et al., 2001; Tan et al., 2007).

It is reasonable to believe that ASIC3 is sensing chemical/metabolic changes in and around the ischemic myocardium after infarction and, thereby, triggering reflexes that contribute to ventricular remodeling. During ischemia, the interstitial pH, which is where cardiac afferent sensory nerve terminals lie, drops from 7.4 to ~7.0 after coronary occlusion. pH drops as small as 7.4 to 7.2 will cause sustained activation of ASIC3 currents within isolated cardiac afferents and generate action potentials (Matasic et al., 2021; Yagi et al., 2006)–and as such, is particularly poised to sense these subtle pH changes within the heart during ischemia. Chronic dilated cardiomyopathy, even in the absence of significant epicardial coronary stenosis, is associated with regional and global myocardial ischemia due to microcirculatory abnormalities and increases in oxygen demand due to increases in wall stress (Juilliere et al., 1993; van den Heuvel et al., 2000). We have previously shown that ASICs within cardiac sympathetic afferents are heteromeric channels composed of the ASIC3 and ASIC2a subunits. When ASIC3 is genetically deleted, the remaining ASIC2a forms homomeric channels that are insensitive to pH changes (Hattori et al., 2009). Thus, loss of ASIC3 renders the cardiac afferent less capable of sensing metabolic changes within the heart.

Besides serving as local pH/metabolic sensors with cardiac afferents, ASICs might also serve as systemic pH sensors. As such, we found that ASICs function as pH sensors within glomus cells of the carotid body, which are the major peripheral chemoreceptors (Tan et al., 2007). Interestingly, the carotid body chemoreflex is augmented in a genetic rat model of hypertension, which correlated with an increase in ASIC3 expression and acid‐evoked currents recorded from glomus cells (Tan et al., 2010). An exaggerated carotid body chemoreflex also contributes to sympathoexcitation in heart failure, and ablation of the carotid bodies in animal models of heart failure improves cardiac function and survival (Ponikowski et al., 2001; Schultz et al., 2013).

ASICs are also expressed in arterial baroreceptors where they contribute to baroreflex sensitivity. We previously found that genetic deletion of ASIC2 in mice was associated with a diminishment of mechanical‐induced depolarization in isolated cultured baroreceptor nodose neurons, a decrease in aortic depressor nerve activity to changes in arterial pressure in anesthetized mice, and diminished baroreflex gain and engagement in conscious mice (Lu et al., 2009). ASIC3 is also expressed in baroreceptors where it colocalizes with ASIC2 (Lu et al., 2009). Given the predilection of different ASIC subunits to heteromultimerize with each other (Benson et al., 2002), it is intriguing to speculate that ASIC3 subunits might also contribute to baroreceptive function. However, our results showed no difference in baroreceptor‐heart rate and baroreceptor‐RSNA reflex sensitivities between ASIC3^−/−^ and WT sham mice. In contrast, after MI, ASIC3^−/−^ mice had decreased baroreceptor‐RSNA reflex sensitivity compared to WT mice (Figure 6c). Various autonomic reflexes are intricately integrated and influence each other in different ways (Abboud & Benson, 2015; Katayama et al., 2019; Persson, 1996). It is possible that ASICs are playing a role in cardiac remodeling through activation of several different sensory pathways, and future studies targeting ASICs within these specific sensory systems are likely to be insightful.

### 4.2 Cardiac remodeling

The degree of cardiac remodeling, and in particular LV dilation, is consistently one of the best predictors of poor clinical outcomes after MI (Azevedo et al., 2016; Pfeffer & Pfeffer, 1987; White et al., 1987). Therapeutics aimed to minimize and even reverse remodeling have shown to improve the symptoms of heart failure and improve survival (Konstam et al., 2003). Both groups of animals, WT and ASIC3^−/−^, experienced remodeling after infarction. However, the characteristics of remodeling differed between the groups. WT mice developed LV dilation, without an increase in LV wall thickness. In contrast, ASIC3^−/−^ mice after MI experienced less LV chamber dilation per IZ fraction and had a significant increase in LV mass and LV thickness. These differential remodeling patterns after MI may explain the increase in LV stroke volume that was observed in ASIC3^−/−^, but not in WT. An increase in hypertrophy associated with increase in wall thickness is often triggered by an increase in LV afterload, such as in the setting of hypertension or aortic stenosis. We found that MAP was similar between MI groups over a 48‐h period; however, ASIC3^−/−^ mice had slightly higher BPV compared to WT mice after MI. We previously described that an increase in BPV generated in mice by sinoaortic baroreceptor denervation activates mechanosensitive pathways, including p125 focal adhesion kinase (p125‐FAK) and p38 mitogen‐activated protein kinase (p38‐MAPK), resulting in cardiac hypertrophy, even though the average level of arterial BP was unchanged compared to sham‐denervated mice (Martinka et al., 2005). We postulated that higher BPV causes more periods of higher‐than‐average BP that triggers LV hypertrophy, and these periods of higher BP are not offset by periods of lower‐than‐normal BP. The baroreceptor denervation model of increased BPV was associated with increased collagen deposition within the LV at 12 weeks following baroreceptor denervation (Martinka et al., 2005), whereas, in our current study we did not find an increase in collagen mRNA at 3 weeks following MI. Perhaps the 3‐week time point following MI was too early for a detectable increase in collagen mRNA expression to occur. Our results also contrast with other findings showing ASIC3 to be protective against fibrosis in a model of isoproterenol‐induced myocardial ischemia (Cheng et al., 2011). Another potential clue to explain the genotype differences in LV remodeling is that ASIC3^−/−^ MI mice had an increase in LV atrial natriuretic peptide that was not found in WT mice after MI. Local expression of atrial natriuretic peptide within the heart associates with LV hypertrophy (Ellmers et al., 2002).

### 4.3 Autonomic nervous system changes in mice after MI

Myocardial infarction triggers a cascade of neurohormonal responses that resemble evolutionarily protective responses to hemorrhage or dehydration. In the latter cases the teleological “goal” is restoration of hemodynamic homeostasis. Along with an increase in sympathetic tone, MI can also lead to a decrease in parasympathetic tone. To our surprise, we found that WT mice did not develop the expected dysautonomia associated with MI. Injection of propranolol in conscious mice and SNA recordings in anesthetized mice did not demonstrate an increase in sympathetic tone following MI in WT mice. Similarly, we did not see a decrease in parasympathetic tone after atropine injection or in measurement of HRV. While mouse models have provided a robust platform to investigate cardiovascular disease, it is critical to understand the unique aspects of mouse physiology as they contrast to larger animal models and humans. In particular, the murine autonomic nervous system is characterized by a relatively high resting sympathetic tone and a lower parasympathetic tone, which has been shown to blunt the effects of sympathoexcitation after MI seen in other mammals (Just et al., 2000; Pizzo et al., 2022; Shusterman et al., 2002).

As alluded to in the previous paragraph, there are limitations in the current study. First, the mouse model used here may not have offered the best animal model for evaluating autonomic changes following MI. A logical next step of this work will be to modulate ASIC channels in an alternative animal model of MI. Alternatively, it is also possible that changes occurred before evaluation of the autonomic nervous system. Although there are limited data on autonomic changes following MI in mice, some data suggest that changes are not observed within the first week post‐MI (Pizzo et al., 2022). However, a longitudinal study to evaluate autonomic changes over time would be informative. Second, infarct size was not histologically confirmed. The goal of this study was to quantify the cardiac remodeling that occurs beyond the initial injury to the myocardium that is shown to occur in the first 3 days following MI (Lindsey et al., 2021). To do this in vivo, echocardiography was used to visualize the akinetic zone. A more thorough investigation of the role of ASICs on infarct size after MI would be interesting. Finally, while we focused on the role of the autonomic nervous system after MI as the mechanism for differential remodeling, ASIC3 has also been implicated to play a role in inflammation (Gregory et al., 2016; Hayashi et al., 2025), and the role of inflammation as a driver of cardiac remodeling after MI is increasingly appreciated (Westman et al., 2016).

Previous work has shown the potential important contribution of cardiac afferents to disadvantageous cardiac remodeling after MI, and ablation of these sensory fibers, using a toxin (resiniferatoxin) targeting TRPV1 channels improved remodeling after infarction (Wang et al., 2017; Wang, Wang, et al., 2014). While the clinical use of resiniferatoxin in a variety of clinical conditions is currently being investigated, the practicality of an invasive procedure involving the application of the toxin to the epicardial surface of the heart in patients suffering from MI is of question. A simpler strategy would be to find a molecular receptor within these sensory neurons that is activated after infarction. Here we demonstrate that genetic loss of ASIC3, a proton‐activated ion channel principally expressed in sensory neurons, alters LV remodeling after MI in mice potentially by decreasing baroreflex sensitivity and increasing BPV. These results provide a strong rationale for follow‐up studies investigating ASIC3 as a potential molecular target to treat post‐MI ventricular remodeling.

## AUTHOR CONTRIBUTIONS

**Karley M. Monaghan**, **David D. Gibbons**, **Chad C. Ward**, **Harald M. Stauss**, and **Christopher J. Benson**: Conceived and designed research. **Karley M. Monaghan**, **David D. Gibbons**, **Chad C. Ward**, **Maram El‐Geneidy**, **William J. Kutschke**, **Kathy A. Zimmerman**, **Donald A. Morgan**, **Anne Marie S. Harding**, **Michelle C. M. Bader**, **Rasna Sabharwal**, and **Robert M. Weiss:** Performed experiments. **Karley M. Monaghan**, **David D. Gibbons**, **Chad C. Ward**, and **Donald A. Morgan**: Analyzed data. **Karley M. Monaghan**, **Anne Marie S. Harding**, **Peter M. Snyder, Robert M. Weiss**, **Kamal Rahmouni**, **Harald M. Stauss**, and **Christopher J. Benson**: Interpreted results of experiments. **Karley M. Monaghan** and **Christopher J. Benson**: Prepared figures, drafted manuscript, and edited and revised manuscript. **Karley M. Monaghan**, **David D. Gibbons**, **Chad C. Ward**, **Maram El‐Geneidy**, **William J. Kutschke**, **Kathy A. Zimmerman**, **Donald A. Morgan**, **Anne Marie S. Harding**, **Michelle C. M. Bader**, **Peter M. Snyder**, **Rasna Sabharwal**, **Robert M. Weiss**, **Kamal Rahmouni**, **Harald M. Stauss**, and **Christopher J. Benson**: Approved final version of manuscript.

## FUNDING INFORMATION

This study was supported by the Department of Veterans Affairs Merit Award (5I01BX000776). Dr. Weiss acknowledges grant support from NIH grants: R01HL171197, R01HL142935, S10OD038119. Dr. Rahmouni is supported by NIH (R01 HL162773 and R01 HL172944), VA (I01 BX004249 and IK6 BX006040), and the University of Iowa Fraternal Order of Eagles Diabetes Research Center. Dr. Sabharwal is supported by NIH (R01 HL149677 and R21 AG070188).

## CONFLICT OF INTEREST STATEMENT

The authors declare no conflicts of interest.

## Supporting information


**Table S1.** Cardiac remodeling as measured by the difference in echocardiographic data measured between 48 h and 3 weeks, separated by sex.

**Table S2.** Body Weights (g) measure at 3 weeks after myocardial infarction (MI) or sham surgery.

**Table S3.** Hemodynamics measured over 48 h at 3 weeks after myocardial infarction (MI) or sham surgery.

## Data Availability

Analyses and datasets of the study are not available publicly but are available from the corresponding author on reasonable request.

## References

[phy270823-bib-0001] Abboud, F. M. , & Benson, C. J. (2015). ASICs and cardiovascular homeostasis. Neuropharmacology, 94, 87–98. 25592213 10.1016/j.neuropharm.2014.12.017 PMC4472389

[phy270823-bib-0002] Allen, N. J. , & Attwell, D. (2002). Modulation of ASIC channels in rat cerebellar Purkinje neurons by ischaemia‐related signals. The Journal of Physiology, 543, 521–529. 12205186 10.1113/jphysiol.2002.020297 PMC2290513

[phy270823-bib-0003] Azevedo, P. S. , Polegato, B. F. , Minicucci, M. F. , Paiva, S. A. , & Zornoff, L. A. (2016). Cardiac remodeling: Concepts, clinical impact, pathophysiological mechanisms and pharmacologic treatment. Arquivos Brasileiros de Cardiologia, 106, 62–69. 26647721 10.5935/abc.20160005 PMC4728597

[phy270823-bib-0004] Baudrie, V. , Laude, D. , & Elghozi, J. L. (2007). Optimal frequency ranges for extracting information on cardiovascular autonomic control from the blood pressure and pulse interval spectrograms in mice. American Journal of Physiology. Regulatory, Integrative and Comparative Physiology, 292, R904–R912. 17038438 10.1152/ajpregu.00488.2006

[phy270823-bib-0005] Benson, C. J. , Eckert, S. P. , & McCleskey, E. W. (1999). Acid‐evoked currents in cardiac sensory neurons: A possible mediator of myocardial ischemic sensation. Circulation Research, 84, 921–928. 10222339 10.1161/01.res.84.8.921

[phy270823-bib-0006] Benson, C. J. , Xie, J. , Wemmie, J. A. , Price, M. P. , Henss, J. M. , Welsh, M. J. , & Snyder, P. M. (2002). Heteromultimers of DEG/ENaC subunits form H+‐gated channels in mouse sensory neurons. Proceedings of the National Academy of Sciences of the United States of America, 99, 2338–2343. 11854527 10.1073/pnas.032678399 PMC122366

[phy270823-bib-0007] Birdsong, W. T. , Fierro, L. , Williams, F. G. , Spelta, V. , Naves, L. A. , Knowles, M. , Marsh‐Haffner, J. , Adelman, J. P. , Almers, W. , Elde, R. P. , & McCleskey, E. W. (2010). Sensing muscle ischemia: Coincident detection of acid and ATP via interplay of two ion channels. Neuron, 68, 739–749. 21092862 10.1016/j.neuron.2010.09.029 PMC3000793

[phy270823-bib-0008] Chatterjee, S. , Biondi‐Zoccai, G. , Abbate, A. , D'Ascenzo, F. , Castagno, D. , Van Tassell, B. , Mukherjee, D. , & Lichstein, E. (2013). Benefits of beta blockers in patients with heart failure and reduced ejection fraction: Network meta‐analysis. BMJ, 346, f55. 23325883 10.1136/bmj.f55 PMC3546627

[phy270823-bib-0009] Chen, B. , Li, Y. , Jiang, S. , Xie, Y. P. , Guo, A. , Kutschke, W. , Zimmerman, K. , Weiss, R. M. , Miller, F. J. , Anderson, M. E. , & Song, L. S. (2012). Beta‐adrenergic receptor antagonists ameliorate myocyte T‐tubule remodeling following myocardial infarction. FASEB Journal, 26, 2531–2537. 22375019 10.1096/fj.11-199505 PMC3360148

[phy270823-bib-0010] Chen, W. W. , Xiong, X. Q. , Chen, Q. , Li, Y. H. , Kang, Y. M. , & Zhu, G. Q. (2015). Cardiac sympathetic afferent reflex and its implications for sympathetic activation in chronic heart failure and hypertension. Acta Physiologica (Oxford, England), 213, 778–794. 25598170 10.1111/apha.12447

[phy270823-bib-0011] Cheng, C. F. , Chen, I. L. , Cheng, M. H. , Lian, W. S. , Lin, C. C. , Kuo, T. B. , & Chen, C. C. (2011). Acid‐sensing ion channel 3, but not capsaicin receptor TRPV1, plays a protective role in isoproterenol‐induced myocardial ischemia in mice. Circulation Journal, 75, 174–178. 21127383 10.1253/circj.cj-10-0490

[phy270823-bib-0012] Cohn, J. N. , Levine, T. B. , Olivari, M. T. , Garberg, V. , Lura, D. , Francis, G. S. , Simon, A. B. , & Rector, T. (1984). Plasma norepinephrine as a guide to prognosis in patients with chronic congestive heart failure. The New England Journal of Medicine, 311, 819–823. 6382011 10.1056/NEJM198409273111303

[phy270823-bib-0013] Dann, M. M. , Clark, S. Q. , Trzaskalski, N. A. , Earl, C. C. , Schepers, L. E. , Pulente, S. M. , Lennord, E. N. , Annamalai, K. , Gruber, J. M. , Cox, A. D. , Lorenzen‐Schmidt, I. , Seymour, R. , Kim, K. H. , Goergen, C. J. , & Mulvihill, E. E. (2022). Quantification of murine myocardial infarct size using 2‐D and 4‐D high‐frequency ultrasound. American Journal of Physiology. Heart and Circulatory Physiology, 322, H359–H372. 34995167 10.1152/ajpheart.00476.2021 PMC8836752

[phy270823-bib-0014] Ellmers, L. J. , Knowles, J. W. , Kim, H. S. , Smithies, O. , Maeda, N. , & Cameron, V. A. (2002). Ventricular expression of natriuretic peptides in Npr1(−/−) mice with cardiac hypertrophy and fibrosis. American Journal of Physiology. Heart and Circulatory Physiology, 283, H707–H714. 12124219 10.1152/ajpheart.00677.2001 PMC4321891

[phy270823-bib-0015] Gao, X. M. , Dart, A. M. , Dewar, E. , Jennings, G. , & Du, X. J. (2000). Serial echocardiographic assessment of left ventricular dimensions and function after myocardial infarction in mice. Cardiovascular Research, 45, 330–338. 10728353 10.1016/s0008-6363(99)00274-6

[phy270823-bib-0016] Gregory, N. S. , Brito, R. G. , Fusaro, M. , & Sluka, K. A. (2016). ASIC3 is required for development of fatigue‐induced hyperalgesia. Molecular Neurobiology, 53, 1020–1030. 25577172 10.1007/s12035-014-9055-4 PMC4499332

[phy270823-bib-0017] Hattori, T. , Chen, J. , Harding, A. M. , Price, M. P. , Lu, Y. , Abboud, F. M. , & Benson, C. J. (2009). ASIC2a and ASIC3 heteromultimerize to form pH‐sensitive channels in mouse cardiac dorsal root ganglia neurons. Circulation Research, 105, 279–286. 19590043 10.1161/CIRCRESAHA.109.202036 PMC4472433

[phy270823-bib-0018] Hayashi, K. , Lesnak, J. B. , Plumb, A. N. , Janowski, A. J. , Rasmussen, L. A. , Vignes, H. , Flanagan, R. , Berardi, G. , Paradee, W. J. , & Sluka, K. A. (2025). Acid‐sensing ion channel 3 in macrophages, but not sensory neurons, mediates development of activity‐induced muscle pain. Brain, Behavior, and Immunity, 130, 106122. 41027498 10.1016/j.bbi.2025.106122

[phy270823-bib-0019] Heidenreich, P. A. , Bozkurt, B. , Aguilar, D. , Allen, L. A. , Byun, J. J. , Colvin, M. M. , Deswal, A. , Drazner, M. H. , Dunlay, S. M. , Evers, L. R. , Fang, J. C. , Fedson, S. E. , Fonarow, G. C. , Hayek, S. S. , Hernandez, A. F. , Khazanie, P. , Kittleson, M. M. , Lee, C. S. , Link, M. S. , … Yancy, C. W. (2022). 2022 AHA/ACC/HFSA guideline for the Management of Heart Failure: A report of the American College of Cardiology/American Heart Association joint committee on clinical practice guidelines. Circulation, 145, e895–e1032. 35363499 10.1161/CIR.0000000000001063

[phy270823-bib-0020] Hill, J. A. , Karimi, M. , Kutschke, W. , Davisson, R. L. , Zimmerman, K. , Wang, Z. , Kerber, R. E. , & Weiss, R. M. (2000). Cardiac hypertrophy is not a required compensatory response to short‐term pressure overload. Circulation, 101, 2863–2869. 10859294 10.1161/01.cir.101.24.2863

[phy270823-bib-0021] Huang, W. , Rubinstein, J. , Prieto, A. R. , Thang, L. V. , & Wang, D. H. (2009). Transient receptor potential vanilloid gene deletion exacerbates inflammation and atypical cardiac remodeling after myocardial infarction. Hypertension, 53, 243–250. 19114647 10.1161/HYPERTENSIONAHA.108.118349 PMC2669745

[phy270823-bib-0022] Huang, W. , Rubinstein, J. , Prieto, A. R. , & Wang, D. H. (2010). Enhanced postmyocardial infarction fibrosis via stimulation of the transforming growth factor‐beta‐Smad2 signaling pathway: Role of transient receptor potential vanilloid type 1 channels. Journal of Hypertension, 28, 367–376. 19887954 10.1097/HJH.0b013e328333af48

[phy270823-bib-0023] Hutchins, G. M. , & Bulkley, B. H. (1978). Infarct expansion versus extension: Two different complications of acute myocardial infarction. The American Journal of Cardiology, 41, 1127–1132. 665522 10.1016/0002-9149(78)90869-x

[phy270823-bib-0024] Iismaa, S. E. , Li, M. , Kesteven, S. , Wu, J. , Chan, A. Y. , Holman, S. R. , Calvert, J. W. , Haq, A. U. , Nicks, A. M. , Naqvi, N. , Husain, A. , Feneley, M. P. , & Graham, R. M. (2018). Cardiac hypertrophy limits infarct expansion after myocardial infarction in mice. Scientific Reports, 8, 6114. 29666426 10.1038/s41598-018-24525-6 PMC5904135

[phy270823-bib-0025] Immke, D. C. , & McCleskey, E. W. (2001). Lactate enhances the acid‐sensing Na+ channel on ischemia‐sensing neurons. Nature Neuroscience, 4, 869–870. 11528414 10.1038/nn0901-869

[phy270823-bib-0026] Jasti, J. , Furukawa, H. , Gonzales, E. B. , & Gouaux, E. (2007). Structure of acid‐sensing ion channel 1 at 1.9 a resolution and low pH. Nature, 449, 316–323. 17882215 10.1038/nature06163

[phy270823-bib-0027] Juilliere, Y. , Marie, P. Y. , Danchin, N. , Gillet, C. , Paille, F. , Karcher, G. , Bertrand, A. , & Cherrier, F. (1993). Radionuclide assessment of regional differences in left ventricular wall motion and myocardial perfusion in idiopathic dilated cardiomyopathy. European Heart Journal, 14, 1163–1169. 8223729 10.1093/eurheartj/14.9.1163

[phy270823-bib-0028] Just, A. , Faulhaber, J. , & Ehmke, H. (2000). Autonomic cardiovascular control in conscious mice. American Journal of Physiology. Regulatory, Integrative and Comparative Physiology, 279, R2214–R2221. 11080088 10.1152/ajpregu.2000.279.6.R2214

[phy270823-bib-0029] Katayama, P. L. , Castania, J. A. , Fazan, R., Jr. , & Salgado, H. C. (2019). Interaction between baroreflex and chemoreflex in the cardiorespiratory responses to stimulation of the carotid sinus/nerve in conscious rats. Autonomic Neuroscience, 216, 17–24. 30598121 10.1016/j.autneu.2018.12.001

[phy270823-bib-0030] Konstam, M. A. , Udelson, J. E. , Anand, I. S. , & Cohn, J. N. (2003). Ventricular remodeling in heart failure: A credible surrogate endpoint. Journal of Cardiac Failure, 9, 350–353. 14583894 10.1054/j.cardfail.2003.09.001

[phy270823-bib-0031] Kostuk, W. J. , Kazamias, T. M. , Gander, M. P. , Simon, A. L. , & Ross, J., Jr. (1973). Left ventricular size after acute myocardial infarction. Serial changes and their prognostic significance. Circulation, 47, 1174–1179. 4267843 10.1161/01.cir.47.6.1174

[phy270823-bib-0032] Leffler, A. , Monter, B. , & Koltzenburg, M. (2006). The role of the capsaicin receptor TRPV1 and acid‐sensing ion channels (ASICS) in proton sensitivity of subpopulations of primary nociceptive neurons in rats and mice. Neuroscience, 139, 699–709. 16515841 10.1016/j.neuroscience.2005.12.020

[phy270823-bib-0033] Lei, L. , Zhou, R. , Zheng, W. , Christensen, L. P. , Weiss, R. M. , & Tomanek, R. J. (2004). Bradycardia induces angiogenesis, increases coronary reserve, and preserves function of the postinfarcted heart. Circulation, 110, 796–802. 15302788 10.1161/01.CIR.0000138933.85923.36

[phy270823-bib-0034] Lindpaintner, K. , Lu, W. , Neidermajer, N. , Schieffer, B. , Just, H. , Ganten, D. , & Drexler, H. (1993). Selective activation of cardiac angiotensinogen gene expression in post‐infarction ventricular remodeling in the rat. Journal of Molecular and Cellular Cardiology, 25, 133–143. 8474123 10.1006/jmcc.1993.1017

[phy270823-bib-0035] Lindsey, M. L. , Brunt, K. R. , Kirk, J. A. , Kleinbongard, P. , Calvert, J. W. , de Castro Bras, L. E. , DeLeon‐Pennell, K. Y. , Del Re, D. P. , Frangogiannis, N. G. , Frantz, S. , Gumina, R. J. , Halade, G. V. , Jones, S. P. , Ritchie, R. H. , Spinale, F. G. , Thorp, E. B. , Ripplinger, C. M. , & Kassiri, Z. (2021). Guidelines for in vivo mouse models of myocardial infarction. American Journal of Physiology. Heart and Circulatory Physiology, 321, H1056–H1073. 34623181 10.1152/ajpheart.00459.2021 PMC8834230

[phy270823-bib-0036] Lingueglia, E. , de Weille, J. R. , Bassilana, F. , Heurteaux, C. , Sakai, H. , Waldmann, R. , & Lazdunski, M. (1997). A modulatory subunit of acid sensing ion channels in brain and dorsal root ganglion cells. The Journal of Biological Chemistry, 272, 29778–29783. 9368048 10.1074/jbc.272.47.29778

[phy270823-bib-0037] Lu, Y. , Ma, X. , Sabharwal, R. , Snitsarev, V. , Morgan, D. , Rahmouni, K. , Drummond, H. A. , Whiteis, C. A. , Costa, V. , Price, M. , Benson, C. , Welsh, M. J. , Chapleau, M. W. , & Abboud, F. M. (2009). The ion channel ASIC2 is required for baroreceptor and autonomic control of the circulation. Neuron, 64, 885–897. 20064394 10.1016/j.neuron.2009.11.007 PMC2807410

[phy270823-bib-0038] Malliani, A. , Schwartz, P. J. , & Zanchetti, A. (1969). A sympathetic reflex elicited by experimental coronary occlusion. The American Journal of Physiology, 217, 703–709. 5807693 10.1152/ajplegacy.1969.217.3.703

[phy270823-bib-0039] Mao, Y. , Zhang, S. , Yu, F. , Li, H. , Guo, C. , & Fan, X. (2015). Ghrelin attenuates liver fibrosis through regulation of TGF‐beta1 expression and autophagy. International Journal of Molecular Sciences, 16, 21911–21930. 26378522 10.3390/ijms160921911 PMC4613288

[phy270823-bib-0040] Martinka, P. , Fielitz, J. , Patzak, A. , Regitz‐Zagrosek, V. , Persson, P. B. , & Stauss, H. M. (2005). Mechanisms of blood pressure variability‐induced cardiac hypertrophy and dysfunction in mice with impaired baroreflex. American Journal of Physiology. Regulatory, Integrative and Comparative Physiology, 288, R767–R776. 15563577 10.1152/ajpregu.00445.2004

[phy270823-bib-0041] Mason, B. N. , Wattiez, A. S. , Balcziak, L. K. , Kuburas, A. , Kutschke, W. J. , & Russo, A. F. (2020). Vascular actions of peripheral CGRP in migraine‐like photophobia in mice. Cephalalgia, 40, 1585–1604. 32811179 10.1177/0333102420949173 PMC7785273

[phy270823-bib-0042] Matasic, D. S. , Holland, N. , Gautam, M. , Gibbons, D. D. , Kusama, N. , Harding, A. M. S. , Shah, V. S. , Snyder, P. M. , & Benson, C. J. (2021). Paradoxical potentiation of acid‐sensing Ion Channel 3 (ASIC3) by amiloride via multiple mechanisms and sites within the channel. Frontiers in Physiology, 12, 750696. 34721074 10.3389/fphys.2021.750696 PMC8555766

[phy270823-bib-0043] Meredith, I. T. , Eisenhofer, G. , Lambert, G. W. , Dewar, E. M. , Jennings, G. L. , & Esler, M. D. (1993). Cardiac sympathetic nervous activity in congestive heart failure. Evidence for increased neuronal norepinephrine release and preserved neuronal uptake. Circulation, 88, 136–145. 8391399 10.1161/01.cir.88.1.136

[phy270823-bib-0044] Minisi, A. J. , & Thames, M. D. (1991). Activation of cardiac sympathetic afferents during coronary occlusion. Evidence for reflex activation of sympathetic nervous system during transmural myocardial ischemia in the dog. Circulation, 84, 357–367. 2060106 10.1161/01.cir.84.1.357

[phy270823-bib-0045] Pan, H. L. , Longhurst, J. C. , Eisenach, J. C. , & Chen, S. R. (1999). Role of protons in activation of cardiac sympathetic C‐fibre afferents during ischaemia in cats. Journal of Physiology, 518(3), 857–866. 10420020 10.1111/j.1469-7793.1999.0857p.x PMC2269450

[phy270823-bib-0046] Persson, P. B. (1996). Modulation of cardiovascular control mechanisms and their interaction. Physiological Reviews, 76, 193–244. 8592729 10.1152/physrev.1996.76.1.193

[phy270823-bib-0047] Pfeffer, J. M. , Pfeffer, M. A. , Fletcher, P. J. , & Braunwald, E. (1991). Progressive ventricular remodeling in rat with myocardial infarction. The American Journal of Physiology, 260, H1406–H1414. 2035662 10.1152/ajpheart.1991.260.5.H1406

[phy270823-bib-0048] Pfeffer, M. A. (1998). ACE inhibitors in acute myocardial infarction: Patient selection and timing. Circulation, 97, 2192–2194. 9631866 10.1161/01.cir.97.22.2192

[phy270823-bib-0049] Pfeffer, M. A. , & Pfeffer, J. M. (1987). Ventricular enlargement and reduced survival after myocardial infarction. Circulation, 75, IV93–IV97. 2952370

[phy270823-bib-0050] Pizzo, E. , Berrettoni, S. , Kaul, R. , Cervantes, D. O. , Di Stefano, V. , Jain, S. , Jacobson, J. T. , & Rota, M. (2022). Heart rate variability reveals altered autonomic regulation in response to myocardial infarction in experimental animals. Frontiers in Cardiovascular Medicine, 9, 843144. 35586660 10.3389/fcvm.2022.843144 PMC9108187

[phy270823-bib-0051] Ponikowski, P. , Chua, T. P. , Anker, S. D. , Francis, D. P. , Doehner, W. , Banasiak, W. , Poole‐Wilson, P. A. , Piepoli, M. F. , & Coats, A. J. (2001). Peripheral chemoreceptor hypersensitivity: An ominous sign in patients with chronic heart failure. Circulation, 104, 544–549. 11479251 10.1161/hc3101.093699

[phy270823-bib-0052] Price, M. P. , McIlwrath, S. L. , Xie, J. , Cheng, C. , Qiao, J. , Tarr, D. E. , Sluka, K. A. , Brennan, T. J. , Lewin, G. R. , & Welsh, M. J. (2001). The DRASIC cation channel contributes to the detection of cutaneous touch and acid stimuli in mice. Neuron, 32, 1071–1083. 11754838 10.1016/s0896-6273(01)00547-5

[phy270823-bib-0053] Protti, A. , Dong, X. , Sirker, A. , Botnar, R. , & Shah, A. M. (2012). MRI‐based prediction of adverse cardiac remodeling after murine myocardial infarction. American Journal of Physiology. Heart and Circulatory Physiology, 303, H309–H314. 22636680 10.1152/ajpheart.00208.2012 PMC3423156

[phy270823-bib-0054] Roberts, C. S. , Maclean, D. , Braunwald, E. , Maroko, P. R. , & Kloner, R. A. (1983). Topographic changes in the left ventricle after experimentally induced myocardial infarction in the rat. The American Journal of Cardiology, 51, 872–876. 6829445 10.1016/s0002-9149(83)80147-7

[phy270823-bib-0055] Rouabhi, M. , Guo, D. F. , Morgan, D. A. , Zhu, Z. , Lopez, M. , Zingman, L. , Grobe, J. L. , & Rahmouni, K. (2021). BBSome ablation in SF1 neurons causes obesity without comorbidities. Molecular Metabolism, 48, 101211. 33722691 10.1016/j.molmet.2021.101211 PMC8065214

[phy270823-bib-0056] Salah, H. M. , Gupta, R. , Hicks, A. J., 3rd , Mahmood, K. , Haglund, N. A. , Bindra, A. S. , Antoine, S. M. , Garcia, R. , Yehya, A. , Yaranov, D. M. , Patel, P. P. , Feliberti, J. P. , Rollins, A. T. , Rao, V. N. , Letarte, L. , Raje, V. , Alam, A. H. , Raval, N. Y. , Howard, B. , & Fudim, M. (2025). Baroreflex function in cardiovascular disease. Journal of Cardiac Failure, 31, 117–126. 39341547 10.1016/j.cardfail.2024.08.062

[phy270823-bib-0057] Schultz, H. D. , Marcus, N. J. , & Del Rio, R. (2013). Role of the carotid body in the pathophysiology of heart failure. Current Hypertension Reports, 15, 356–362. 23824499 10.1007/s11906-013-0368-x PMC3801176

[phy270823-bib-0058] Shusterman, V. , Usiene, I. , Harrigal, C. , Lee, J. S. , Kubota, T. , Feldman, A. M. , & London, B. (2002). Strain‐specific patterns of autonomic nervous system activity and heart failure susceptibility in mice. American Journal of Physiology. Heart and Circulatory Physiology, 282, H2076–H2083. 12003814 10.1152/ajpheart.00917.2001

[phy270823-bib-0059] Stauss, H. M. (2007). Power spectral analysis in mice: What are the appropriate frequency bands? American Journal of Physiology. Regulatory, Integrative and Comparative Physiology, 292, R902–R903. 17038437 10.1152/ajpregu.00716.2006

[phy270823-bib-0060] Stauss, H. M. , Morgan, D. A. , Anderson, K. E. , Massett, M. P. , & Kregel, K. C. (1997). Modulation of baroreflex sensitivity and spectral power of blood pressure by heat stress and aging. The American Journal of Physiology, 272, H776–H784. 9124438 10.1152/ajpheart.1997.272.2.H776

[phy270823-bib-0061] Sutherland, S. P. , Benson, C. J. , Adelman, J. P. , & McCleskey, E. W. (2001). Acid‐sensing ion channel 3 matches the acid‐gated current in cardiac ischemia‐sensing neurons. Proceedings of the National Academy of Sciences of the United States of America, 98, 711–716. 11120882 10.1073/pnas.011404498 PMC14653

[phy270823-bib-0062] Sutton, M. G. , & Sharpe, N. (2000). Left ventricular remodeling after myocardial infarction: Pathophysiology and therapy. Circulation, 101, 2981–2988. 10869273 10.1161/01.cir.101.25.2981

[phy270823-bib-0063] Tan, Z. Y. , Lu, Y. , Whiteis, C. A. , Benson, C. J. , Chapleau, M. W. , & Abboud, F. M. (2007). Acid‐sensing ion channels contribute to transduction of extracellular acidosis in rat carotid body glomus cells. Circulation Research, 101, 1009–1019. 17872465 10.1161/CIRCRESAHA.107.154377

[phy270823-bib-0064] Tan, Z. Y. , Lu, Y. , Whiteis, C. A. , Simms, A. E. , Paton, J. F. , Chapleau, M. W. , & Abboud, F. M. (2010). Chemoreceptor hypersensitivity, sympathetic excitation, and overexpression of ASIC and TASK channels before the onset of hypertension in SHR. Circulation Research, 106, 536–545. 20019330 10.1161/CIRCRESAHA.109.206946 PMC2846115

[phy270823-bib-0065] Uchida, Y. , & Murao, S. (1975). Acid‐induced excitation of afferent cardiac sympathetic nerve fibers. American Journal of Physiology, 228, 27–33. 1080016 10.1152/ajplegacy.1975.228.1.27

[phy270823-bib-0066] van den Heuvel, A. F. , Veldhuisen, D. J. , Wall, E. E. , Blanksma, P. K. , Siebelink, H. M. , Vaalburg, W. M. , van Gilst, W. H. , & Crijns, H. J. (2000). Regional myocardial blood flow reserve impairment and metabolic changes suggesting myocardial ischemia in patients with idiopathic dilated cardiomyopathy. Journal of the American College of Cardiology, 35, 19–28. 10636254 10.1016/s0735-1097(99)00499-4

[phy270823-bib-0067] Wang, H. , Leinwand, L. A. , & Anseth, K. S. (2014). Roles of transforming growth factor‐beta1 and OB‐cadherin in porcine cardiac valve myofibroblast differentiation. FASEB Journal, 28, 4551–4562. 25008089 10.1096/fj.14-254623 PMC4202096

[phy270823-bib-0068] Wang, H. J. , Rozanski, G. J. , & Zucker, I. H. (2017). Cardiac sympathetic afferent reflex control of cardiac function in normal and chronic heart failure states. The Journal of Physiology, 595, 2519–2534. 28116751 10.1113/JP273764 PMC5390865

[phy270823-bib-0069] Wang, H. J. , Wang, W. , Cornish, K. G. , Rozanski, G. J. , & Zucker, I. H. (2014). Cardiac sympathetic afferent denervation attenuates cardiac remodeling and improves cardiovascular dysfunction in rats with heart failure. Hypertension, 64, 745–755. 24980663 10.1161/HYPERTENSIONAHA.114.03699 PMC4162756

[phy270823-bib-0070] Wang, W. , & Zucker, I. H. (1996). Cardiac sympathetic afferent reflex in dogs with congestive heart failure. American Journal of Physiology, 271, R751–R756. 8853400 10.1152/ajpregu.1996.271.3.R751

[phy270823-bib-0071] Wemmie, J. A. , Price, M. P. , & Welsh, M. J. (2006). Acid‐sensing ion channels: Advances, questions and therapeutic opportunities. Trends in Neurosciences, 29, 578–586. 16891000 10.1016/j.tins.2006.06.014

[phy270823-bib-0072] Westman, P. C. , Lipinski, M. J. , Luger, D. , Waksman, R. , Bonow, R. O. , Wu, E. , & Epstein, S. E. (2016). Inflammation as a driver of adverse left ventricular remodeling after acute myocardial infarction. Journal of the American College of Cardiology, 67, 2050–2060. 27126533 10.1016/j.jacc.2016.01.073

[phy270823-bib-0073] White, H. D. , Norris, R. M. , Brown, M. A. , Brandt, P. W. , Whitlock, R. M. , & Wild, C. J. (1987). Left ventricular end‐systolic volume as the major determinant of survival after recovery from myocardial infarction. Circulation, 76, 44–51. 3594774 10.1161/01.cir.76.1.44

[phy270823-bib-0074] Yadav, S. , Ninh, V. K. , Lovelace, J. W. , Ma, J. , Pham, A. , Salamon, R. J. , Ji, E. , Na, Y. , Fu, Z. , Ugochukwu, S. I. , Cui, W. , Sehgal, R. , King, K. R. , & Augustine, V. (2026). A triple‐node heart‐brain neuroimmune loop underlying myocardial infarction. Cell, 189, 800–817. 41605213 10.1016/j.cell.2025.12.058

[phy270823-bib-0075] Yagi, J. , Wenk, H. N. , Naves, L. A. , & McCleskey, E. W. (2006). Sustained currents through ASIC3 ion channels at the modest pH changes that occur during myocardial ischemia. Circulation Research, 99, 501–509. 16873722 10.1161/01.RES.0000238388.79295.4c

[phy270823-bib-0076] Yan, G. X. , & Kleber, A. G. (1992). Changes in extracellular and intracellular pH in ischemic rabbit papillary muscle. Circulation Research, 71, 460–470. 1628400 10.1161/01.res.71.2.460

[phy270823-bib-0077] Zhang, Y. , Taufalele, P. V. , Cochran, J. D. , Robillard‐Frayne, I. , Marx, J. M. , Soto, J. , Rauckhorst, A. J. , Tayyari, F. , Pewa, A. D. , Gray, L. R. , Teesch, L. M. , Puchalska, P. , Funari, T. R. , McGlauflin, R. , Zimmerman, K. , Kutschke, W. J. , Cassier, T. , Hitchcock, S. , Lin, K. , … Abel, E. D. (2020). Mitochondrial pyruvate carriers are required for myocardial stress adaptation. Nature Metabolism, 2, 1248–1264. 10.1038/s42255-020-00288-1 PMC8015649 33106689

