# Supplementary material for: Genetic deletion of ASIC3 alters left ventricular remodeling and autonomic function after myocardial infarction in mice
Source: Physiol Rep. 2026 Mar 11;14(5):e70823. doi: 10.14814/phy2.70823 (PMC12976581; doi:10.14814/phy2.70823)
Supplement: Supplementary file 1 — Table S1. Cardiac remodeling as measured by the difference in echocardiographic data measured between 48 h and 3 weeks, separated by sex. [file PHY2-14-e70823-s003.docx]

Supplemental Table 1. Cardiac remodeling as measured by the difference in echocardiographic data measured between 48 hours and 3 weeks, separated by sex.

|  |  | **WT Sham** | **WT MI** | **ASIC3^-/-^ Sham** | **ASIC3^-/-^ MI** |
| --- | --- | --- | --- | --- | --- |
| LVEDV (uL) | Male | 1.4 ± 4.2 | 29.2 ± 10.0 | -6.8 ± 4.2 | 22.3 ± 15.2 |
|  | Female | 1.6 ± 4.4 | 25.3 ± 9.0 | 2.3 ± 2.4 | 26.9 ± 6.5 |
| LVESV (uL) | Male | -0.3 ± 1.6 | 26.5 ± 9.0 | -1.2 ± 1.2 | 9.3 ± 12.9 |
|  | Female | 1.5 ± 1.8 | 22.3 ± 8.8 | 0.2 ± 0.4 | 18.5 ± 5.1 |
| LVEF | Male | 0.02 ± 0.03 | -0.04 ± 0.03 | -0.02 ± 0.02 | 0.13 ± 0.05 |
|  | Female | -0.02 ± 0.02 | -0.01 ± 0.05 | 0.00 ± 0.02 | 0.01 ± 0.03 |
| LV Mass (mg) | Male | 0.8 ± 4.2 | 0.04 ± 4.2 | -1.1 ± 3.5 | 18.3 ± 5.4 |
|  | Female | -2.3 ± 2.8 | 6.2 ± 3.4 | 9.6 ± 4.8 | 9.8 ± 3.4 |
| SV (uL) | Male | 1.6 ± 3.4 | 2.8 ± 2.2 | -5.6 ± 3.4 | 12.9 ± 5.7 |
|  | Female | 0.06 ± 2.7 | 3.0 ± 3.2 | 2.1 ± 2.2 | 8.4± 2.9 |

Values are means ± SE; Statistical analysis by three-way ANOVA with Tukey post hoc adjustment revealed no statistical differences between the sexes for any of the following parameters: left ventricular end diastolic volume (LVEDV): *F*_(1,111)_ = 0.1419, *P* = 0.7071; left ventricular end systolic volume (LVESV): *F*_(1,111)_ = 0.1280, *P* = 0.7211; left ventricular ejection fraction (LVEF): *F*_(1,111)_ = 01.518, *P* = 0.2205; left ventricular (LV) mass: *F*_(1,111)_ = 0.1742, *P* = 0.6772; stroke volume (SV): *F*_(1,111)_ = 0.03767, *P* = 0.8465; WT Sham male: *N* = 10; WT Sham female: *N* = 11; WT MI male: *N* = 25; WT MI female: *N* = 14; ASIC3^-/-^ Sham male: *N* = 12; ASIC3^-/-^Sham female: *N* = 12; ASIC3^-/-^ MI male: *N* = 9; ASIC3^-/-^MI female: *N* = 26.
