# Supplementary material for: Genetic deletion of ASIC3 alters left ventricular remodeling and autonomic function after myocardial infarction in mice
Source: Physiol Rep. 2026 Mar 11;14(5):e70823. doi: 10.14814/phy2.70823 (PMC12976581; doi:10.14814/phy2.70823)
Supplement: Supplementary file 2 — Table S2. Body Weights (g) measure at 3 weeks after myocardial infarction (MI) or sham surgery. [file PHY2-14-e70823-s002.docx]

Supplemental Table 2. Body Weights (mg) measure at 3 weeks after myocardial infarction (MI) or sham surgery.

|  |  | **WT Sham** | **WT MI** | **ASIC3^-/-^ Sham** | **ASIC3^-/-^ MI** |
| --- | --- | --- | --- | --- | --- |
| Baseline | Total | 20.2 ± 0.7 | 21.3 ± 0.5 | 21.1 ± 1.0 | 18.6 ± 0.7 |
|  | Male | 23.0 ± 0.3 | 23.0 ± 0.4 | 23.9 ± 0.9 | 22.0 ± 0.9 |
|  | Female | 18.3 ± 0.3 | 18.4 ± 0.2 | 18.3 ± 1.1 | 17.5 ± 0.6 |
| 48 Hours | Total | 19.4 ± 0.8 | 20.0 ± 0.5 | 20.6 ± 1.0 | 17.6 ± 0.7 |
|  | Male | 22.4 ± 0.5 | 21.8 ± 0.3 | 23.7 ± 0.7 | 21.5 ± 0.8 |
|  | Female | 17.3 ± 0.3 | 16.9 ± 0.2 | 17.5 ± 0.9 | 16.3 ± 0.5 |
| 3 Weeks | Total | 22.1± 0.8 | 22.8 ± 0.6 | 23.4 ± 1.1 | 20.6 ± 0.7 |
|  | Male | 25.0 ± 0.8 | 24.6 ± 0.4 | 26.6 ± 0.8 | 24.2 ± 1.0 |
|  | Female | 20.0 ± 0.4 | 19.7 ± 0.5 | 20.1 ± 1.2 | 19.3 ± 0.6 |

Values are means ± SE; Statistical analysis by three-way ANOVA with Tukey post hoc adjustment revealed no differences between the groups at each time point. (Total: WT Sham: *N* = 12; WT MI: *N* = 22; ASIC3^-/-^ Sham: *N* = 14; ASIC3^-/-^MI: *N*=20. Male: WT Sham: *N* = 5; WT MI: *N* = 14; ASIC3^-/-^ Sham: *N* = 7; ASIC3^-/-^ MI: *N* = 5. Female: WT Sham: *N* = 7; WT MI: *N* = 8; ASIC3^-/-^Sham: *N* = 7; ASIC3^-/-^ MI: *N* = 15.)
