# Supplementary material for: Genetic deletion of ASIC3 alters left ventricular remodeling and autonomic function after myocardial infarction in mice
Source: Physiol Rep. 2026 Mar 11;14(5):e70823. doi: 10.14814/phy2.70823 (PMC12976581; doi:10.14814/phy2.70823)
Supplement: Supplementary file 3 — Table S3. Hemodynamics measured over 48 h at 3 weeks after myocardial infarction (MI) or sham surgery. [file PHY2-14-e70823-s001.docx]

Supplemental Table 3. Hemodynamics measured over 48 hours at 3 weeks after myocardial infarction (MI) or sham surgery.

|  |  | **WT Sham** | **WT MI** | **ASIC3^-/-^ Sham** | **ASIC3^-/-^ MI** |
| --- | --- | --- | --- | --- | --- |
| **HR (bpm)** | Average | 569 ± 44 | 573 ± 54 | 579 ± 36 | 552 ± 30 |
|  | Day | 534 ± 48 | 538 ± 52 | 543 ± 34 | 509 ± 31 |
|  | Night | 605 ± 42 | 609 ± 58 | 615 ± 39 | 596 ± 32 |
| **SBP (mmHg)** | Average | 117 ± 5 | 106 ± 5 | 118 ± 6 | 117 ± 20 |
|  | Day | 110 ± 3 | 101 ± 4 | 111 ± 6 | 111 ± 25 |
|  | Night | 123 ± 7 | 111 ± 6* | 126 ± 7 | 123 ± 16^#^ |
| **MAP (mmHg)** | Average | 104 ± 3 | 95 ± 4 | 104 ± 5 | 102 ± 15 |
|  | Day | 98 ± 3 | 90 ± 4 | 97 ± 5 | 96 ± 20 |
|  | Night | 111 ± 6 | 100 ± 5* | 112 ± 7 | 108 ± 12 |
| **DBP (mmHg)** | Average | 92 ± 3 | 82 ± 4* | 91 ± 8 | 88 ± 12 |
|  | Day | 86 ± 4 | 77 ± 4 | 84 ± 8 | 81 ± 17 |
|  | Night | 98 ± 4 | 88 ± 5* | 97 ± 8 | 94 ± 9 |

Continuous recording of heart rate (HR), systolic blood pressure (SBP), mean arterial pressure (MAP), and diastolic blood pressure (DBP) measured at 3 weeks after myocardial infarction (MI) or sham surgery over a 48-hour period. Values are the average of the average (full 48 hours), day (24 hours of light period), and night (24 hours of dark periods). Values are means ± SD; Statistical analysis by two-way ANOVA with Fisher’s LSD post hoc adjustment was performed for each variable. No significant effects or statistical differences were found in HR. No significant effects or statistical differences were observed in the average or day SBP. Significant effects for genotype (*F*_(1,28)_ = 4.57, *P* = 0.041) and surgery (*F*_(1,28)_ = 4.52, *P* = 0.043) were seen for the SBP at night with differences between WT Sham and WT MI (*P* = 0.017) and WT MI and ASIC3^-/-^ MI (*P* = 0.017). No significant effects were found for the average or day MAP. A significant surgery effect was seen for the MAP at night (*F*_(1,28)_ = 6.43, *P* = 0.017) with a significant difference between WT Sham and WT MI (*P* = 0.009) No significant effects were found for the day DBP. A significant surgery effect was seen for the average DBP (*F*_(1,28)_ = 5.55, *P* = 0.026) with a difference between WT Sham and WT MI (*P* = 0.018). A significant surgery effect was seen for the DBP at night (*F*_(1,28)_ = 8.23, *P* = 0.008) with a significant difference between WT Sham and WT MI (*P* = 0.005). (WT Sham: *N* = 8; WT MI: *N* = 8; ASIC3^-/-^ Sham: *N* = 8; ASIC3^-/-^MI: *N* = 8). * *P* < 0.05 for comparisons between MI groups and their respective shams. ^#^ *P* <0.05 for comparisons between WT MI and ASIC3^-/-^MI.
